# Chromosome-level genome assembly and annotation of the loquat (*Eriobotrya japonica*) genome

**DOI:** 10.1093/gigascience/giaa015

**Published:** 2020-03-06

**Authors:** Shuang Jiang, Haishan An, Fangjie Xu, Xueying Zhang

**Affiliations:** Forestry and Pomology Research Institute, Shanghai Key Lab of Protected Horticultural Technology, Shanghai Academy of Agricultural Sciences, Shanghai 201403, China

**Keywords:** loquat, genome assembly, Nanopore, Hi-C

## Abstract

**Background:**

The loquat (*Eriobotrya japonica*) is a species of flowering plant in the family Rosaceae that is widely cultivated in Asian, European, and African countries. It blossoms in the winter and ripens in the early summer. The genome of loquat has to date not been published, which limits the study of molecular biology in this cultivated species. Here, we used the third-generation sequencing technology of Nanopore and Hi-C technology to sequence the genome of *Eriobotrya*.

**Findings:**

We generated 100.10 Gb of long reads using Oxford Nanopore sequencing technologies. Three types of Illumina high-throughput sequencing data, including genome short reads (47.42 Gb), transcriptome short reads (11.06 Gb), and Hi-C short reads (67.25 Gb), were also generated to help construct the loquat genome. All data were assembled into a 760.1-Mb genome assembly. The contigs were mapped to chromosomes by using Hi-C technology based on the contacts between contigs, and then a genome was assembled exhibiting 17 chromosomes and a scaffold N50 length of 39.7 Mb. A total of 45,743 protein-coding genes were annotated in the *Eriobotrya* genome, and we investigated the phylogenetic relationships between the *Eriobotrya* and 6 other Rosaceae species. *Eriobotrya* shows a close relationship with *Malus* and *Pyrus*, with the divergence time of *Eriobotrya* and *Malus* being 6.76 million years ago. Furthermore, chromosome rearrangement was found in *Eriobotrya* and *Malus*.

**Conclusions:**

We constructed the first high-quality chromosome-level *Eriobotrya* genome using Illumina, Nanopore, and Hi-C technologies. This work provides a valuable reference genome for molecular studies of the loquat and provides new insight into chromosome evolution in this species.

## Data Description

### Background

The *Eriobotrya* L. is a flowering plant in the family Rosaceae [1], including ∼25 species identified by most taxonomists. Sixteen of the species are native to China [2]. Cultivated loquats in Asia mainly belong to *Eriobotrya japonica* (NCBI:txid32224). The loquat originated from China and has been produced widely throughout other Asian countries (Japan and Korea), some southern European countries (Turkey, Italy, and France), and several northern African countries (Morocco and Algeria) [3]. This species is a large evergreen tree that is grown commercially for its yellow or red fruit. The loquat, apple, pear, and peach are closely related [4]. In contrast, the maturity period of the loquat is early summer, which is earlier in the year than most other cultivated fruits. The loquat is evergreen and blossoms in winter. After flower bud differentiation, the loquat blossoms without a long period of dormancy. The loquat exhibits infinite inflorescences, and 1 inflorescence produces many fruits, which increases its ability to adapt to low winter temperatures.

In the present study, we generated a genome assembly for the loquat with 17 chromosomes and a genome size of 760 Mb. The genome assembly was created using Nanopore long reads and high-throughput chromosome conformation capture (Hi-C) data. Illumina paired-end sequence was used for the base and indel correction. The completeness and continuity of the genome were comparable to those of other important Rosaceae species. The high-quality reference genome generated in this study will facilitate research on population genetic traits and functional gene identification related to important characteristics of the loquat.

### Sample collection

**Figure 1:**
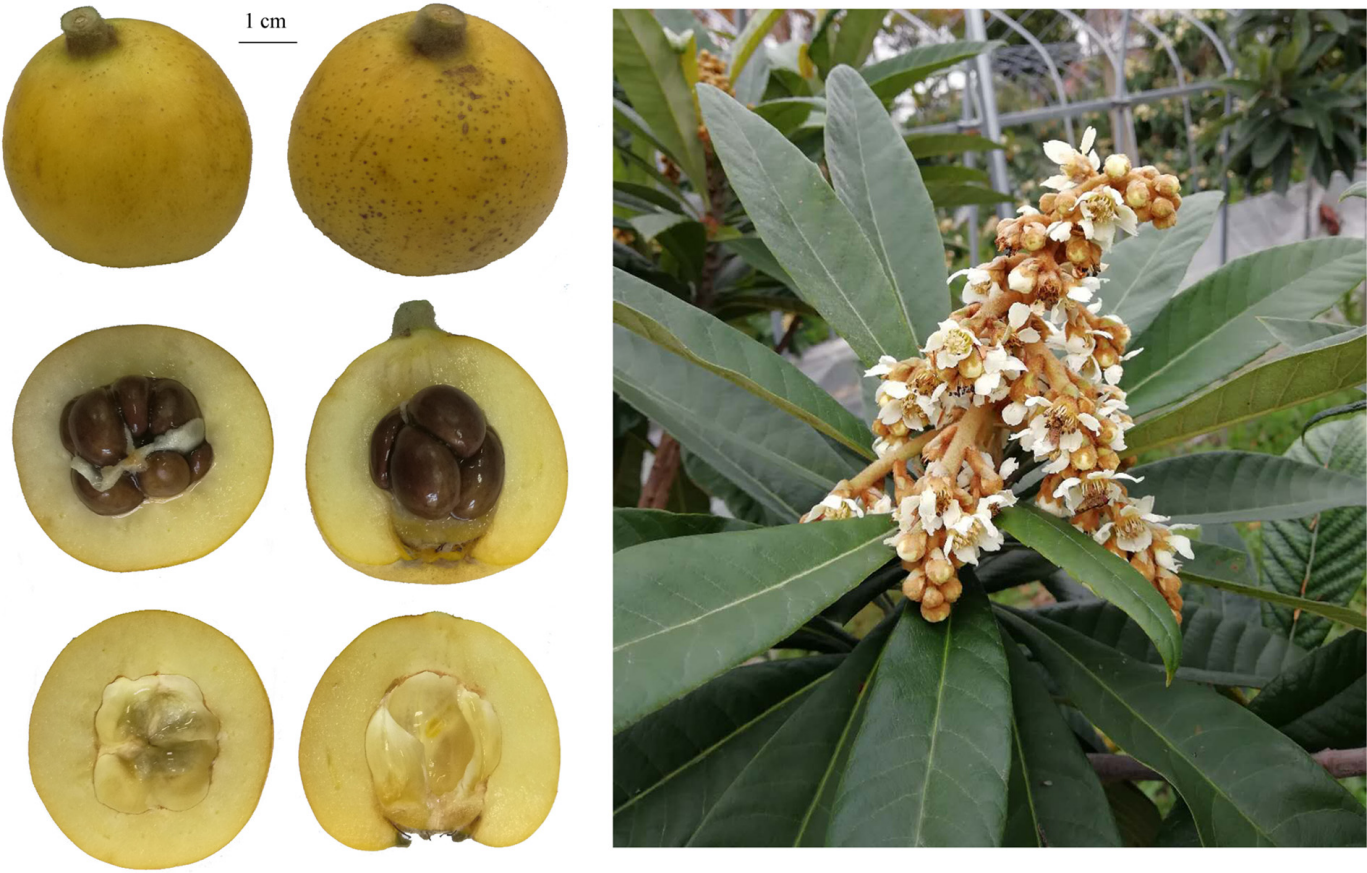
Picture of a loquat variety, Seventh Star (*Eriobotrya japonica*).

*Eriobotrya japonica* cv. Seventh Star is a cultivar bred by the team of Dr. Xueying Zhang at the Shanghai Academy of Agricultural Sciences (SAAS), Shanghai, China (Fig. 1), that is widely cultivated in Shanghai. Young leaves were collected from an individual of Seventh Star on 20 March 2019 at the experimental farm of SAAS in Zhuanghang Town (Fengxian, Shanghai, China). This tree was 14 years old and was considered to be in the adult phase. The leaves were frozen in liquid nitrogen and stored at −80°C until DNA extraction. Total genomic DNA was extracted from the leaf tissues following the CTAB protocol [5]. The leaves, fruit, buds, roots, and branches were collected for RNA extraction via the CTAB-LiCl method.

### Estimation of genome size and heterozygosity analysis

The qualified genomic DNA was randomly disrupted by ultrasonic oscillation to generate the fragments of 350 bp, and then a small fragment sequencing library was constructed by terminal repair, the addition of A bases and linkers, target fragment selection, and PCR. The library was subjected to paired-end 150 bp (PE 150) sequencing using the Illumina HiSeq 4000 platform (Illumina HiSeq 4000 System, RRID:SCR_016386). The data were subjected to quality control and used for analysis. The results showed that a total of 47.42 Gb of data were obtained (Table 1). Ten thousand reads were randomly selected to search the Nucleotide Sequence Database in the NCBI using BLAST, and 90.62% of the reads were mapped to the *Malus* and *Pyrus* genomes. No reads were mapped to microorganisms or animals, which confirmed that the sample was free from contamination. The guanine-cytosine content of the genome is estimated to be ∼39.65%.

**Table 1:** Sequencing data used for loquat genome assembly and annotation

Sequencing type	Platform	Library size (bp)	Clean data (Gb)	Application
Genome short reads	Illumina HiSeq 4000	350	47.42	Genome survey and assessment
Nanopore reads	Nanopore platform	20,000	100.10	Contig assembly
Hi-C reads	Illumina HiSeq 4000	300–700	67.25	Chromosome construction
Transcriptome short reads	Illumina HiSeq 4000	200–500	11.06	Genome annotation and assessment

A *k-*mer is an oligonucleotide sequence of length *k* extracted from the sliding windows of sequencing data. Under the premise of a uniform distribution of sequencing reads, the following formula is obtained:

$$\begin{eqnarray*}
{\rm{Genomic\ size}} &=& \frac{{\rm{total\, number\, of\, bases}}}{{\rm{average\, sequencing\, depth}}} \nonumber\\
&=& \frac{{\rm{total\, }}k{\rm{mer}}}{{\rm{median\, }}k{\rm{mer\, depth}}}.
\end{eqnarray*}$$

**Figure 2:**
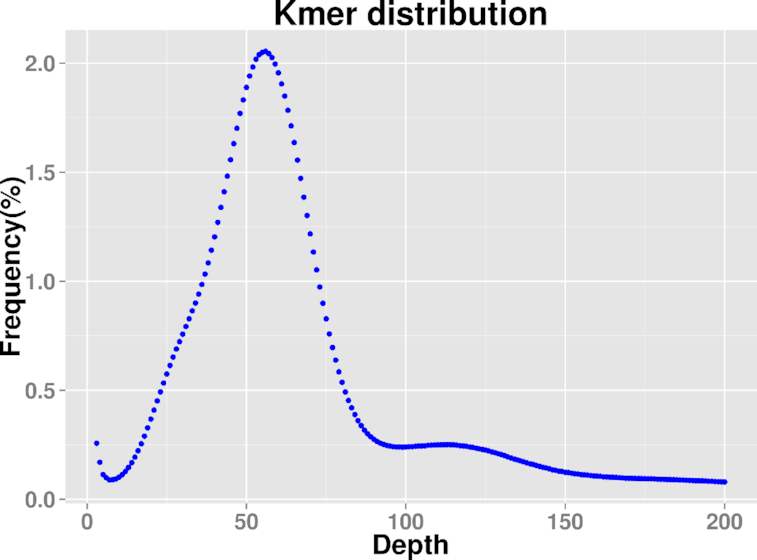
The *k-*mer analysis (*k* = 23) of *Eriobotrya japonica* genome characteristics.

A *k-*mer map of *k* = 21 was constructed using the 350-bp library data (Fig. 2) for the evaluation of genome size, the repeat sequence ratio, and heterozygosity. The main peak corresponding to the *k-*mer depth was 55, which was the average *k-*mer depth. A sequence in which the *k-*mer depth appeared to be more than twice the depth of the main peak (depth value, 111) was considered a repeat sequence. A *k-*mer depth was half of the main peak (depth value, 27.5), indicating that the sequence was heterozygous. The total number of *k-*mers obtained from the sequencing data was 41,072,179,362. After the removal of *k-*mers with an abnormal depth, a total of 39,711,658,265 *k-*mers were used for genome size estimation, and the calculated genome length was ∼710.83 Mb, which was consistent with the size of 654.40 Mb estimated by means of flow cytometry [6]. According to the *k-*mer distribution, the estimated repeat sequence ratio was ∼54.56%. There was no obvious heterozygous peak, and the heterozygosity was low, at 0.48%.

### Nanopore, Hi-C, and RNA sequencing

Genomic DNA was extracted and sequenced following the instructions of the Ligation Sequencing Kit (Nanopore, Oxfordshire, UK). The DNA was purified, and its quantity was assessed with a Qubit 2.0 Fluorometer (Thermo Fisher, CA, USA). The DNA was randomly sheared, and fragments of ∼20 kb were enriched and purified. Damaged DNA and ends were enzymatically repaired with the NEBNext End Repair/dA-Tailing Module (NEB, MA, UK). Then, a 20-kb library was constructed and sequenced on the Nanopore PromethION platform using 2low cells, according to the manufacturer's protocols (PromethION, RRID:SCR_017987). Approximately 106.23 Gb of data were obtained. After data quality control, the final data volume was 100.10 Gb (Table 1). A Hi-C sample library was constructed from genomic DNA from the fresh leaves of the loquat [7]. The main procedures included cross-linking the DNA, restriction enzyme digestion, end repair, DNA cyclization, and DNA purification. The library was sequenced on the Illumina HiSeq 4000 platform [8]. A total of 67.25 Gb of clean data were obtained, and the Q30 was 94.38%. RNA sequencing (RNA-seq) samples were obtained by mixing equal amounts of RNA extracted from each tissue (leaf, fruit, bud, root, and branch) and used for library construction. After sequencing on the Illumina HiSeq 4000 platform, we obtained 11.06 Gb of sequencing data (Table 1).

### Genome assembly based on Nanopore and Hi-C data

In Nanopore sequencing data, the N50 value and the average length of the reads reached 18.06 and 16.15 kb, respectively (Additional Table S1). According to the estimated genome size (710.83 Mb), the sequencing depth was 131.69×. First, the Nanopore reads were corrected by the correction function in Canu v1.4 (Canu, RRID:SCR_015880) [9]. Second, the corrected reads (6,198,187 reads) were assembled by SMARTdenovo (SMARTdenovo, RRID:SCR_017622) [10] to obtain the draft genome with 597 contigs covering 732.25 Mb. Third, Racon (Racon, RRID:SCR_017642) [11] was used to calibrate the draft genome with Nanopore reads through 3 rounds, and the genome size was corrected to 753.38 Mb. Fourth, Pilon v1.21 (Pilon, RRID:SCR_014731) [12] was used to calibrate the draft genome with short genome reads from the Illumina HiSeq 4000 platform through 3 rounds with error radio of 1.64%, 0.07%, and 0.01%, respectively. Finally, the total length of the draft genome sequence was 760.10 Mb, composed of 597 contigs, and the contig N50 was 5.02 Mb.

BWA (BWA, RRID:SCR_010910, v0.7.15) [13] was used to map the Hi-C short reads obtained from the Illumina HiSeq platform against the draft genome. The comparison mode was “aln,” and the other parameters were set to the defaults. The number of unique mapped read pairs was 135,734,826, which accounted for 60.42% of the total read pairs. These unique read pairs were evaluated by HiC-Pro (HiC-Pro, RRID:SCR_017643) [14] to compare the valid interaction pairs and the invalid interaction pairs mapped to the draft genome. The result showed that the proportion of valid interaction pairs was 73.97%. In conclusion, the Hi-C library exhibited high quality. The contigs were split at a length of 50 kb and reassembled according to Hi-C data. A position that could not be restored to the original assembly sequence was listed as a candidate error region, and the low Hi-C coverage depth in this region confirmed this error. After correction, 819 contigs (760.10 Mb) were identified. LACHESIS (LACHESIS, RRID:SCR_017644) [15] was used to group, sort, and orient all contigs. A total of 800 contigs (757.53 Mb, 99.66%) could be mapped to 17 chromosomes. In the assembly process, the order and direction of 305 contigs were clear, accounting for 676.24 Mb (88.97%), which were assembled to the chromosomes (Additional Table S2). Finally, 17 chromosomes and 514 unplaced scaffolds were obtained in the chromosome-level genome (Table 2). The scaffold N50 was 39.7 Mb.

**Table 2:** Assembly statistics

	Software	Assembly level	No. of sequences	N50 (Mb)	size (Gb)
Nanopore	Smartdenovo, Racon, and Pilon	Contig	597	5.0	760.1
Nanopore and Hi-C	Lachesis	Chromosome	17 + 514^a^	39.7	676.2 + 83.9

a There are 514 unplaced scaffolds in the final chromosome-level assembly. These unplaced contigs comprise ∼10.73% of total bases in the genome assembly size.

### Evaluation of assembly quality

The integrity of the assembled genome was assessed. First, BWA v0.7.15 (BWA, RRID:SCR_010910) [13] was used to compare the short reads obtained from the Illumina HiSeq sequencing data with the reference genome. The percent of reads mapped to the reference genome was up to 99.69%. Second, CEGMA v2.5 (CEGMA, RRID:SCR_015055) [16] was used to assess the integrity of 458 conserved core genes for eukaryotes, and 451 (98.47%) genes were present in the assembled genome. Third, the BUSCO database v2.0 (BUSCO, RRID:SCR_015008) [17] was used to assess the completeness of gene regions, which contained 1,440 conserved core genes. The results showed that 96.81% of the plant single-copy orthologues were complete. Complete single-copy and multicopy genes accounted for 64.65% and 32.15% of the genes, respectively. These results therefore indicated that the loquat genome assembly presented high quality and coverage.

### Genome annotation

LTR_FINDER (LTR_FINDER, RRID:SCR_015247) [18] and RepeatScout (RepeatScout, RRID:SCR_014653) [19] were used for the *de novo* prediction of repetitive sequences in the loquat genome, and all isolated sequences were then classified by PASTEClassifier (PASTEClassifier, RRID:SCR_017645) [20] and mapped to the Repbase database using RepeatMasker (RepeatMasker, RRID:SCR_012954) [21]. A total of 449.72 Mb of repeat sequences were identified, accounting for 59.17% of the genome size (Table 3). Among these repeat sequences, 48.6% (369.44 Mb) and 9.65% (73.34 Mb) were predicted as Class I transposons and Class II retrotransposons (Table 3). In Class I, *copia* and gypsy retrotransposons account for 15.84% (120.38 Mb) and 26.28% (199.73 Mb) of the retrotransposons, respectively. In Class II, terminal inverted repeat (TIR) and helitron transposons account for 6.85% and 1.96% of the transposons, respectively. The results showed that retrotransposons accounted for a large proportion of the loquat genome.

**Table 3:** Repeat sequences in the loquat genome

Type	No.	Length	Rate (%)
Class I	457,393	369,440,909	48.60
Class I/DIRS	11,457	9,761,251	1.28
Class I/LINE	26,529	8,851,756	1.16
Class I/LTR	36,969	15,617,403	2.05
Class I/LTR/Copia	141,908	120,380,193	15.84
Class I/LTR/Gypsy	183,863	199,727,884	26.28
Class I/PLE|LARD	54,589	14,439,960	1.90
Clas I/SINE	812	155,412	0.02
Class I/SINE|TRIM	7	3,188	0
Class I/TRIM	1,223	497,670	0.07
ClassI/Unknown	36	6,192	0
Class II	210,159	73,341,918	9.65
Class II/Crypton	7	403	0
Class II/Helitron	45,852	14,912,320	1.96
Class II/MITE	561	159,816	0.02
ClassII/Maverick	405	107,504	0.01
Class II/TIR	140,384	52,101,491	6.85
Class II/Unknown	22,950	6,060,384	0.80
Potential host gene	2,021	451,961	0.06
SSR	346	66,302	0.01
Unknown	26,488	6,427,210	0.85
Total	669,919	449,728,153	59.17

DIRS: Dictyostelium intermediate repeat sequence; LARD: large retrotransposon derivative; LINE: long interspersed nuclear element; LTR: long terminal repeat; MITE: miniature inverted-repeat transposable element; PLE: Penelope-like element; SINE: short interspersed nuclear element; SSR: simple sequence repeat; TIR: terminal inverted repeat; TRIM: terminal-repeat retrotransposons in miniature.

**Figure 3:**
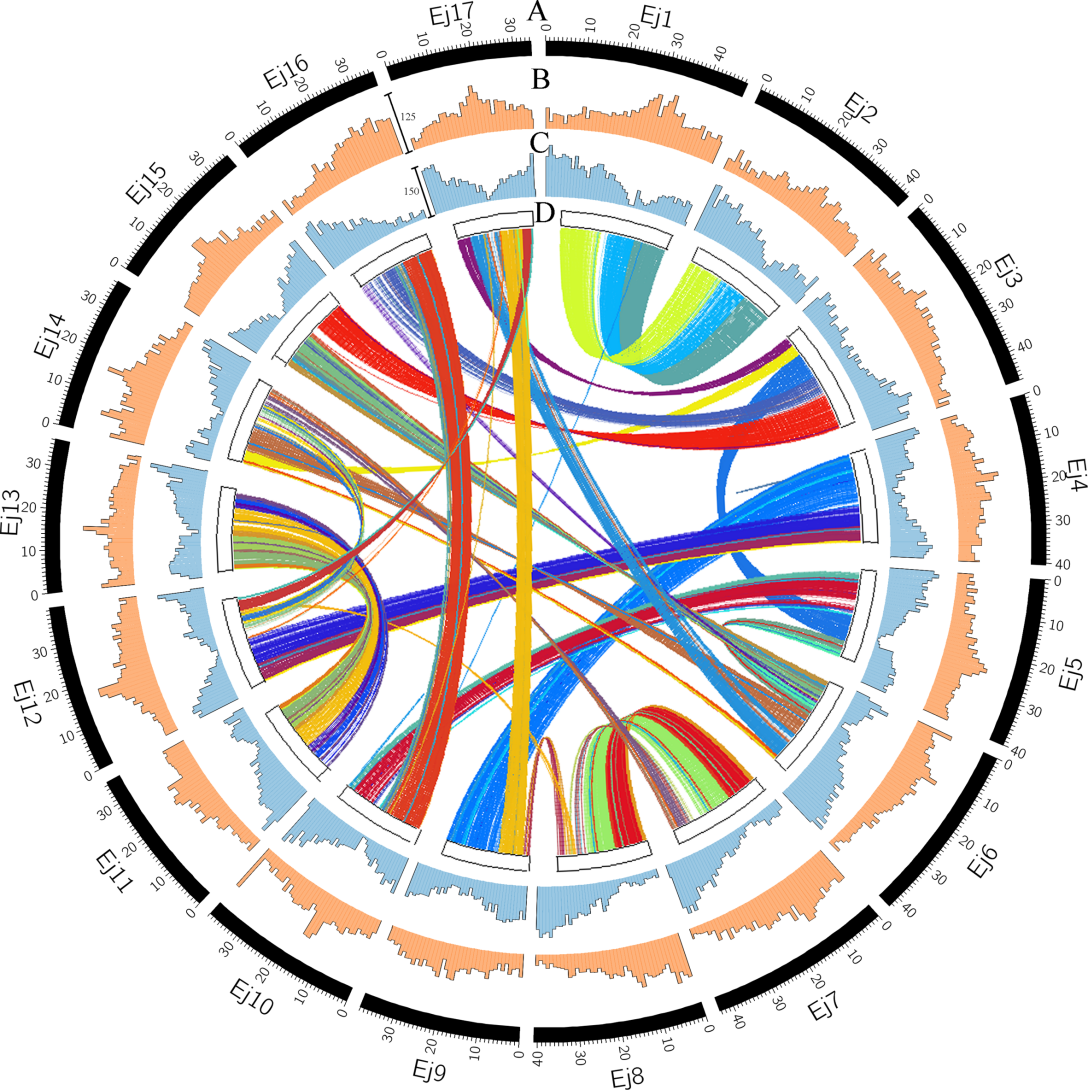
Summary of the *de novo* genome assembly and sequencing analysis of *Eriobotrya japonica*. A, Chromosome number; B, numbers of repeat sequences per megabase pair; C, numbers of protein coding genes per megabase pair; and D, paralogous relationships between E*. japonica* chromosomes.

Protein-coding genes were predicted on the basis of 3 different strategies, including *de novo* prediction, homologous species prediction, and Unigene prediction. Genscan (Genscan, RRID:SCR_012902) [22], Augustus v2.4 (Augustus, RRID:SCR_015981) [23], GlimmerHMM v3.0.4 (GlimmerHMM, RRID:SCR_002654) [24], GeneID v1.4 (GeneID, RRID:SCR_002473) [25], and SNAP (SNAP, RRID:SCR_005501) [26] were used for *de novo* prediction (Additional Table S3). GeMoMa v1.3.1 (GeMoM, RRID:SCR_017646) [27] was used for prediction based on homologous species. The transcripts were assembled by using Hisat v2.0.4 (Hisat, RRID:SCR_015530) [28] and Stringtie v1.2.3 (StringTie, RRID:SCR_016323) [29] with default parameters based on RNA-seq data [30], and then TransDecoder (TransDecoder, RRID:SCR_017647) [31], GeneMarkS-T v5.1 (GeneMarkS-T, RRID:SCR_017648) [32], and PASA v2.0.2 (PASA, RRID:SCR_014656) [33] were used for gene prediction (Additional Table S3). Finally, EvidenceModeler (EVM, RRID:SCR_014659, v1.1.1) [34] was used to integrate the prediction results obtained through the above 3 methods. The Venn diagram showed that 27,685 genes were predicted via all 3 strategies (Additional Fig. S1), and 45,743 genes corresponding to 160.87 Mb were predicted (Additional Table S3). To better understand gene function, we searched all 45,743 protein-coding genes against protein databases, including InterProScan, KEGG, SwissProt, and TrEMBL. The results showed that 98.69% of the genes could be annotated from these databases. The distribution of repetitive sequences and protein-coding genes is shown in Fig. 3B and C.

Based on the Rfam database [35], Blastn (Blastn, RRID:SCR_001598) was used for genome-wide alignment to identify microRNAs (miRNAs) and ribosomal RNAs (rRNAs). Transfer RNAs (tRNAs) were predicted with tRNAscan-SE (tRNAscan-SE, RRID:SCR_010835) [36]. A total of 656 tRNAs, 6,211 rRNAs, and 121 miRNAs were predicted. GeneWise (GeneWise, RRID:SCR_015054) [37] was used to identify immature stop codons and frameshift mutations in the predicted genes to obtain pseudogenes, and 7,642 pseudogenes were obtained.

### Gene clusters and duplication

**Figure 4:**
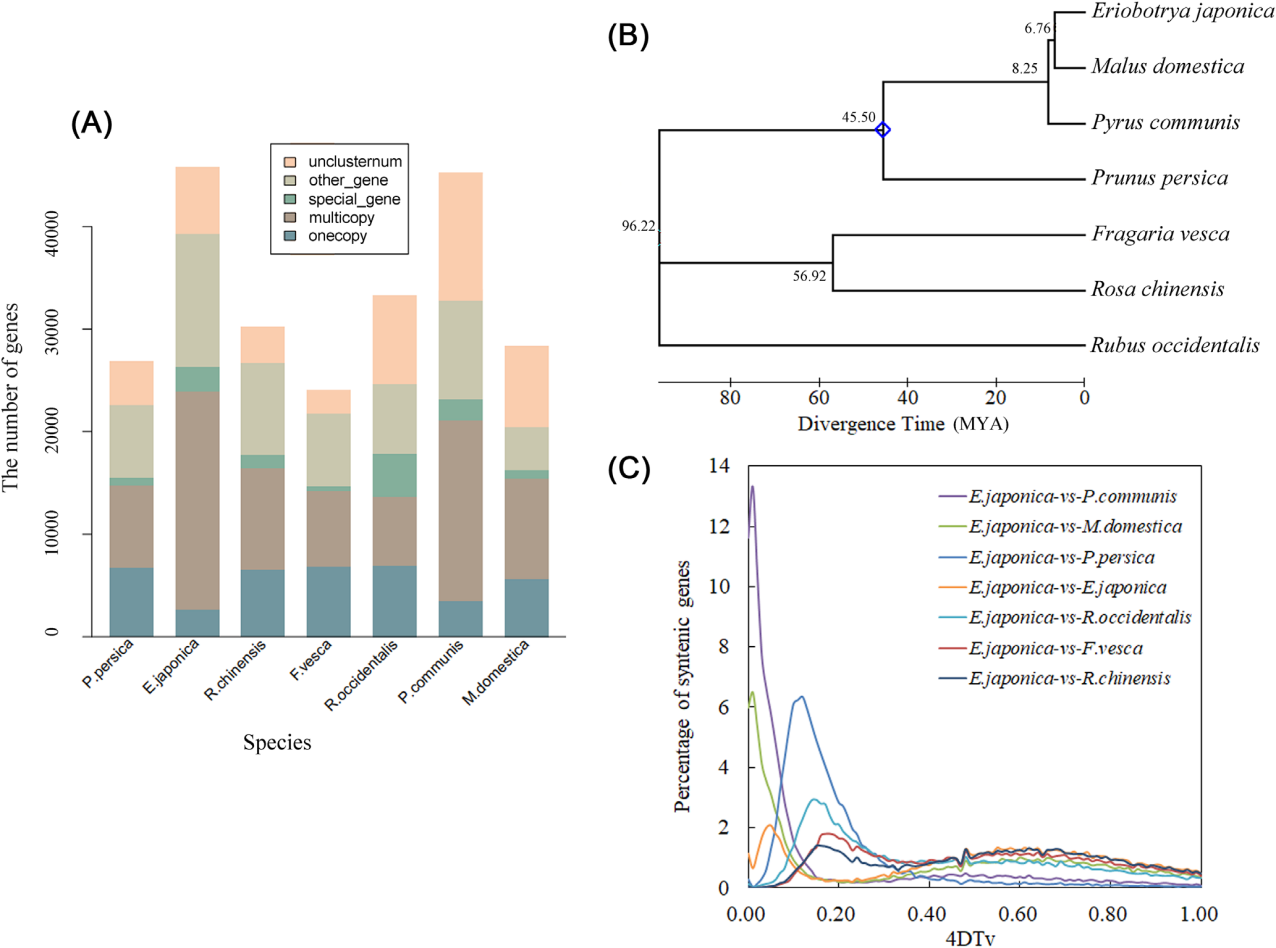
The genome evolution of *Eriobotrya*. (A) Comparison of copy numbers in gene clusters of *Eriobotrya* genomes and 6 related species genomes. Onecopy, single copy genes. Multicopy, multicopy genes. Special_gene, species-specific genes. Other_gene, the rest of the clustered genes other than the above genes. Unclusternum, unclustered genes. (B) Constructed phylogenetic tree and divergence time estimation (MYA, millions of years ago). The blue dot represents the fossil record used in the node.. (C) 4DTv analyses in *Eriobotrya* and related species.

The protein sequences of *E. japonica* and 6 related species (*Malus domestica, Prunus persica, Pyrus communis, Rubus occidentalis, Rosa chinensis*, and *Fragaria vesca*) were compared to analyze the duplication of genes and the classification of species-specific genes between species. The genomes of all related species were downloaded from the Genome Database for Rosaceae. OrthoMCL (OrthoMCL, RRID:SCR_007839) [38] was used to identify the gene families unique to all species. In *E. japonica*, 45,743 genes were grouped into 17,333 gene families (Table 4), which was a greater number than in the other species. The number of genes and gene families in *E. japonica* was similar to that in *P. communis*, which exhibited 45,217 genes and 16,875 gene families. *E. japonica* presented 665 unique families, suggesting that these families were special in the loquat genome. The classification of genes showed that the number of single-copy genes in loquat was lower than in the other species, and 1,849 single-copy genes were identified. The loquat and pear presented large numbers of multiple-copy genes (Fig. 4A). CAFE (CAFE, RRID:SCR_005983) was used to study gene family expansion [39]. The results showed that 182 genes were expanded in *E. japonica* compared with *M. domestica* and *P. communis*, including the NB-ARC domain, transposase family tnp2, and the Myb/SANT-like DNA-binding domain (Additional Table S4).

**Table 4:** Statistics of gene family classification in 7 species

Species	Total genes	Cluster No.	Total family	Unique family
*E. japonica*	45,743	39,294	17,333	665
*M. domestica*	28,306	20,426	12,797	365
*P. communis*	45,217	32,764	16,875	819
*P. persica*	26,873	22,583	14,969	310
*R. occidentalis*	33,253	24,641	15,479	1,241
*F. vesca*	24,034	21,789	14,859	196
*R. chinensis*	30,214	26,705	15,326	473

Owing to limited computing power, 51 single-copy genes in loquat and 6 related species were randomly selected to construct a phylogenetic tree using MEGA (MEGA, RRID:SCR_000667, v7.0.26). The method of maximum-likelihood–based phylogenetic analyses was performed with *R. occidentalis* as the outgroup. The results indicated that *Eriobotrya* shows a close relationship with *Malus* and *Pyrus* (Fig. 4). To further investigate the divergence times of these species, the RelTime model was used. Fossil records were downloaded from the TIMETREE website [40] and used to calibrate the results. The divergence time of *Malus* and *Prunus* was set to 45.50 million years ago. The results showed that the loquat diverged from *Malus* ∼6.76 million years ago (Fig. 4B).

4DTv (4-fold degenerate synonymous sites of the third codons) values were calculated according to the homologous gene pairs between 2 species or within the species itself. The 4DTv distribution map revealed 2 whole-genome replication events. A divergence peak value (4DTv ∼ 0.01) was observed for *E. japonica* vs *P. communis* in the map, and low values were found in *E. japonica* vs *R. chinensis* (Fig. 4C), which suggested that the divergence of *E. japonica* and *P. communis* occurred later than the divergence of *E. japonica* and *R. chinensis*. In a self-alignment of the chromosomes based on gene synteny, a peak value (0.05) was found among the 4DTv values, suggesting that a whole-genome or large-fragment duplication occurred in the *Eriobotrya* genome. *Eriobotrya* and *Malus* presented clear 2:2 synteny, implying that they shared a common whole-genome duplication event.

### Chromosome evolution between the *Malus, Prunus*, and *Eriobotrya* genomes

**Figure 5:**
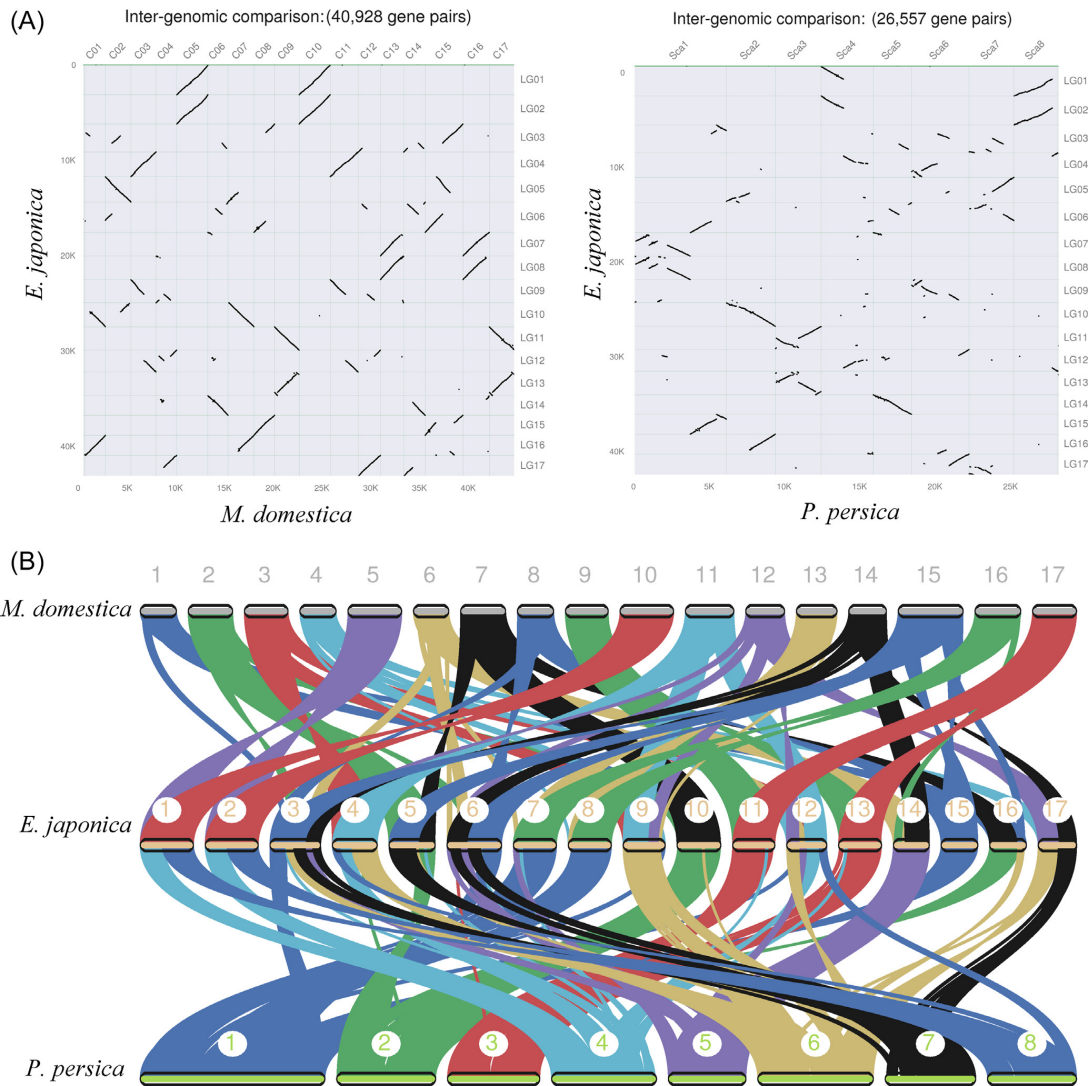
The chromosomes collinearity among *Malus, Prunus*, and *Eriobotrya*. (A) The inter-genomic comparison. (B) The chromosome map in 3 species.

The evolution of the *Eriobotrya* chromosomes and gene collinearity was evaluated using MCScan v0.8 (MCScan, RRID:SCR_017650). The chromosomes of *Prunus* and *Malus* were used as reference genomes. A total of 26,557 and 40,928 gene pairs were found in the inter-genomic comparisons of *Eriobotrya* vs *Prunus* and *Eriobotrya* vs *Malus*, respectively. The alignments of syntenic chromosomes were visualized between *Malus, Prunus*, and *Eriobotrya* (Fig. 5A). There were fewer scattered points in *Eriobotrya* vs *Malus* than in *Eriobotrya* vs *Prunus*, suggesting a close relationship between *Eriobotrya* and *Malus*. The frequency of large-scale fragment rearrangements was found among *Malus, Prunus*, and *Eriobotrya*, including inversions and translocations (Fig. 5B). In the comparison of *Prunus* and *Eriobotrya*, the Sac1, 4, and 8 chromosomes of *Prunus* were found to be duplicated (Fig. 5A). Sac1 was divided into LG07/LG08 and LG06/LG15 in *Eriobotrya*. Sac4 and Sac8 were combined and formed LG01 and LG02. Sac5 was not duplicated and formed LG14 in *Eriobotrya*, suggesting that the other copy of Sac5 was lost in the whole-genome duplication. In the comparison of *Malus* and *Eriobotrya*, C05 and C10 in *Malus* were combined and formed LG01 and LG02 in *Eriobotrya*. C09 and C17 formed LG11 and LG13. This result suggested that fragment rearrangements occurred widely on the chromosomes of *Malus* and *Eriobotrya*. These findings implied that *Malus, Prunus*, and *Eriobotrya* shared some chromosome regions and that extensive chromosome rearrangements occurred. Overall, these findings provide new insight into the evolution of *Eriobotrya* chromosomes.

## Conclusion

To our knowledge, this is the first report of the chromosome-level genome assembly of *E. japonica* using the third-generation sequencing technology of Nanopore and Hi-C. A total of 45,743 high-quality protein-coding genes were annotated by integrating the results from 3 different methods, including *de novo* prediction, homologous species prediction, and Unigene prediction. Phylogenetic analysis indicated that *Eriobotrya* is closely related to *Malus*. The analysis showed that a whole-genome or large-fragment duplication occurred in the *Eriobotrya* genome. The chromosomal rearrangement was found in *Eriobotrya* and *Malus*. This work provides valuable chromosome-level genomic data for loquat and important genomic data for studying loquat traits.

## Availability of Supporting Data and Materials

The raw sequence data have been deposited in NCBI under project accession No. PRJNA579885. The run of clean reads in RNA-seq, Hi-C, Illumina HiSeq, and Nanopore were deposited in the Genome Sequence Archive in NCBI under the Bioproject accession No. PRJNA579885 (SRR10377313– SRR10377316). Data were also submitted to the BIG Data Center, Beijing Institute of Genomics (BIG), Chinese Academy of Sciences, under BioProject No. PRJCA001836. For genome assembly data, the accession No. is GWHAAZU00000000 in the BIG Genome Warehouse. The run of clean reads of Nanopore, Illumina HiSeq, HiC, and RNA-seq data were deposited in the Genome Sequence Archive in BIG under the accession No. CRR078404–CRR078407. All supporting data and materials are available in the *GigaScience* GigaDB database [41].

## Additional Files

**Additional Figure S1:** COG function classification of all unigenes.

**Additional Table S1:** The sequence length of reads in Nanopore.

**Additional Table S2:** The details of the distribution of each chromosome sequence.

**Additional Table S3:** Gene prediction result statistics.

**Additional Table S4:** The number of expansion genes in *E. japonica* compared with *M. domestica* and *P. communis*.

giaa015_GIGA-D-19-00365_Original_Submission

giaa015_GIGA-D-19-00365_Revision_1

giaa015_Response_to_Reviewer_Comments_Original_Submission

giaa015_Reviewer_1_Report_Original_Submission Robert van Buren -- 11/9/2019 Reviewed

giaa015_Reviewer_1_Report_Revision_1 Robert van Buren -- 1/27/2020 Reviewed

giaa015_Reviewer_2_Report_Original_Submission Jian-Feng Mao, Ph.D. -- 11/24/2019 Reviewed

giaa015_Reviewer_3_Report_Original_Submission Soichiro Nagano -- 11/28/2019 Reviewed

giaa015_Supplemental_Figure_and_Tables

## Abbreviations

4DTv: 4-fold degenerate synonymous sites of the third codons; BLAST: Basic Local Alignment Search Tool; bp: base pairs; BUSCO: Benchmarking Universal Single-Copy Orthologs; BWA: Burrows-Wheeler Aligner; CTAB: cetyl trimethylammonium bromide; Gb: gigabase pairs; GO: Gene Ontology; Hi-C: high-throughput chromosome conformation capture; HiSeq: high-throughput sequencing; kb: kilobase pairs; KEGG: Kyoto Encyclopedia of Genes and Genomes; Mb: megabase pairs; miRNA: microRNA; NCBI: National Center for Biotechnology Information; PASA: Program to Assemble Spliced Alignments; RNA-seq: RNA sequencing; rRNA: ribosomal RNA; SAAS: Shanghai Academy of Agricultural Sciences; SNAP: Scalable Nucleotide Alignment Program; TIR: terminal inverted repeat; TrEMBL: a database of translated proteins from European Bioinformatics Institute; tRNA: transfer RNA.

## Competing Interests

The authors declare that they have no competing interests.

## Funding

This work was financed by a Grant for Agriculture Applied Technology Development Program from Shanghai Agriculture Committee (2015-6-2-2) and a Grant from the National Natural Science Foundation of China (No. 31701886).

## Authors' Contributions

S.J. performed the experiments and wrote the manuscript. H.A. helped to collect the samples and revise the manuscript. F.X. helped to analyze the data and revise the manuscript. X.Z. was involved in designing the research and revised the manuscript. All authors read and approved the manuscript.
