# Supplementary material for: Chromosome-level genome assembly and annotation of the loquat (Eriobotrya japonica) genome
Source: Gigascience. 2020 Mar 6;9(3):giaa015. doi: 10.1093/gigascience/giaa015 (PMC7059265; doi:10.1093/gigascience/giaa015)
Supplement: giaa015_GIGA-D-19-00365_Original_Submission [file giaa015_giga-d-19-00365_original_submission.pdf]

GigaScience

The chromosome-level genome assembly and annotation of the loquat (Eriobotrya japonica) genome

--Manuscript Draft--

**Manuscript Number:**	GIGA-D-19-00365
**Full Title:**	The chromosome-level genome assembly and annotation of the loquat (Eriobotrya japonica) genome
**Article Type:**	Data Note
**Funding Information:**	National Natural Science Foundation of China (31701886) — Dr. Shuang Jiang<br>a Grant for Agriculture Applied Technology Development Program from Shanghai Agriculture Committee (2015-6-2-2) — Dr. Xueying Zhang

**Abstract:**	Background<br><br>The loquat (Eriobotrya japonica) is a species of flowering plant in the family Rosaceae, which is widely cultivated in Asian, European, and African countries. It flowered in the winter and ripen in the early summer. The genome of loquat was still not reported, which limited the study of molecular biology in the loquat. Here we used third-generation sequencing Nanopore and High-through chromosome conformation capture (Hi-C) technology to sequence the genome of the Eriobotrya to provide the reference to the researchers.Findings<br><br>We generated 100.10 Gb long reads using Nanopore sequencing technologies. Three Illumina high-throughput sequencing data, including Genome short reads (47.42 Gb), transcriptome short reads (11.06 Gb) and Hi-C short reads (67.25 Gb) were also sequenced to construct the loquat genome. All data were assembled into a 760.1 Mb genome assembly. The Hi-C technology assembled contigs into chromosomes based on the contacts between contigs and then assembled a genome with 17 chromosomes and a scaffold N50 length of 39.7 Mb. A total of 45,743 protein-coding genes were annotated in the Eriobotrya genome, and we analyzed phylogenetic relationships between the Eriobotrya and the other six Rosaceae species. The Eriobotrya has a close relationship with Malus and Pyrus, and the divergence time of Eriobotrya and Malus was 6.76 million years ago. Furthermore, the chromosome rearrangement was found in Eriobotrya and Malus. Conclusions: We constructed the first high-quality chromosome-level Eriobotrya genome using Illumina, Nanopore, and Hi-C technologies. This work provides a valuable reference genome for the molecular studies of the loquat, and give a new insight of chromosome evolution in the loquat.

**Corresponding Author:**	Xueying Zhang<br><br>CHINA
**Corresponding Author Secondary Information:**	
**Corresponding Author's Institution:**	
**Corresponding Author's Secondary Institution:**	
**First Author:**	Shuang Jiang
**First Author Secondary Information:**	
**Order of Authors:**	Shuang Jiang<br>Haishan An<br>Fangjie Xu

Powered by Editorial Manager® and ProduXion Manager® from Aries Systems Corporation

	Xueying Zhang
**Order of Authors Secondary Information:**	
**Additional Information:**	
**Question**	**Response**
Are you submitting this manuscript to a special series or article collection?	No
**Experimental design and statistics**<br><br>Full details of the experimental design and statistical methods used should be given in the Methods section, as detailed in our Minimum Standards Reporting Checklist. Information essential to interpreting the data presented should be made available in the figure legends.<br><br>Have you included all the information requested in your manuscript?	Yes
**Resources**<br><br>A description of all resources used, including antibodies, cell lines, animals and software tools, with enough information to allow them to be uniquely identified, should be included in the Methods section. Authors are strongly encouraged to cite Research Resource Identifiers (RRIDs) for antibodies, model organisms and tools, where possible.<br><br>Have you included the information requested as detailed in our Minimum Standards Reporting Checklist?	Yes
**Availability of data and materials**<br><br>All datasets and code on which the conclusions of the paper rely must be either included in your submission or deposited in publicly available repositories (where available and ethically appropriate), referencing such data using a unique identifier in the references and in	Yes

the "Availability of Data and Materials" section of your manuscript.<br><br>Have you have met the above requirement as detailed in our Minimum Standards Reporting Checklist?	

Click here to download Manuscript 2019.10.15.docx

The chromosome-level genome assembly and annotation of the loquat (*Eriobotrya japonica*) genome

Shuang Jiang†, Haishan An†, Fangjie Xu, Xueying Zhang*

Forestry and Pomology Research Institute, Shanghai Key Lab of Protected Horticultural Technology, Shanghai Academy of Agricultural Sciences, Shanghai, 201403, China

†Equal contribution.

***Correspondence:** Xueying Zhang; loquat_zhang@126.com

Abstract

Background: The loquat (*Eriobotrya japonica*) is a species of flowering plant in the family Rosaceae, which is widely cultivated in Asian, European, and African countries. It flowered in the winter and ripen in the early summer. The genome of loquat was still not reported, which limited the study of molecular biology in the loquat. Here we used third-generation sequencing Nanopore and High-through chromosome conformation capture (Hi-C) technology to sequence the genome of the *Eriobotrya* to provide the reference to the researchers. **Findings:** We generated 100.10 Gb long reads using Nanopore sequencing technologies. Three Illumina high-throughput sequencing data, including Genome short reads (47.42 Gb), transcriptome short reads (11.06 Gb) and Hi-C short reads (67.25 Gb) were also sequenced to construct the loquat genome. All data were assembled into a 760.1 Mb genome assembly. The Hi-C technology assembled contigs into chromosomes based on the contacts between contigs and then assembled a genome with 17 chromosomes and a scaffold N50 length of 39.7 Mb. A total of 45,743 protein-coding genes were annotated in the *Eriobotrya* genome, and we analyzed phylogenetic relationships between the *Eriobotrya* and the other six Rosaceae species. The *Eriobotrya* has a close relationship with *Malus* and *Pyrus*, and the divergence time of *Eriobotrya* and *Malus* was 6.76 million years ago. Furthermore, the chromosome rearrangement was found in *Eriobotrya* and *Malus*. **Conclusions:** We constructed the first high-quality chromosome-level *Eriobotrya* genome using Illumina, Nanopore, and Hi-C technologies. This work provides a valuable reference genome for the molecular studies of the loquat, and give a new insight of chromosome evolution in the loquat.

Data Description

Background

The genus *Eriobotrya* L. (common name loquat) is a species of flowering plant in the family Rosaceae [1], and about twenty-five species are classified by most taxonomists. Sixteen species were native in China [2]. Cultivated loquats in Asia mainly belong to *Eriobotrya japonica* (NCBI: txid 32224). The loquat was originated from China and has been also produced widely throughout other Asian countries (Japan and Korea), some southern European countries (Turkey, Italy, and France), and several Northern African countries (Morocco and Algeria) [3]. It is a large evergreen tree, grown commercially for its yellow or red fruit. The relationship of loquat, apple, pear, and peach are close [4]. In comparison, the maturity period of the loquat is in early summer, which is earlier than most of the fruits in a year. The loquat is evergreen and blooms in winter. The top buds become flowers. After flower bud differentiation, the loquat flowered without a long period of dormancy. The loquat has infinite inflorescence, and one inflorescence can pick up many fruits, which enhance the ability to adapt to the low temperature in the winter.

In the present study, we present a genome assembly for the loquat with 17 chromosomes and a genome size of 760 Mb. The genome assembly was created using Nanopore long reads and Hi-C data. Illumina paired-end sequence was used for the base and indel correction. The completeness and continuity of the genome were comparable with those of other important Rosaceae species. The high-quality reference genome generated in this study will facilitate research on population genetic traits and functional gene identification related to important characteristics of the loquat.

Sample collection

Eriobotrya japonica cv. Seventh Star is a cultivar bred by the team of Dr. Xueying Zhang in Shanghai Academy of Agricultural Sciences (SAAS, Shanghai, China) (Fig. 1), and it was widely cultivated in Shanghai, China. The young leaves were collected from an individual of Seventh Star on Mar. 20, 2019 at the experimental farm of SAAS in Zhuanghang Town (Fengxian, Shanghai, China). This tree was 14 years old and considered to be in the adult phase. The leaves were frozen in liquid nitrogen and stored at −80 ^{o}C until DNA extraction. Total genomic DNA was extracted from the leaf tissues following the CTAB protocol [5]. The leaf, fruit, bud, root, and branch were collected to RNA extraction.

Estimation of genome size and heterozygosity analysis

The qualified genomic DNA was randomly interrupted by ultrasonic oscillation into the fragment of 350 bp, and then a small fragment sequencing library was constructed by terminal repair, addition of A, addition of linker, target fragment selection, and PCR. The library was subjected to double-end 150 bp (PE 150) sequencing using an Illumina Hiseq 4000. The data was subjected to quality control and used for the analysis. The result showed that a total of 47.42 Gb data were obtained (Table 1). The reads were searched by the NT database, which confirmed that the sample is free from contamination. Evaluation of the chloroplast of the species revealed a very low extra-nuclear DNA content. The GC content of the genome is estimated to be approximately 39.65%.

Kmer is an oligonucleotide sequence of length k extracted from the sliding window of the sequencing data. Under the premise of uniform distribution of sequencing reads, the following formula is obtained:

$$\text{Genomic size} = \frac{\text{total number of bases}}{\text{average sequencing depth}} = \frac{\text{total kmer}}{\text{average kmer depth}}$$

A kmer map of k=21 was constructed using 350 bp library data (Fig. 2) for evaluation of the genome size, repeat sequence ratio, and heterozygosity. The main peak corresponding to the kmer depth is 55, which is the average kmer depth. A sequence in which the kmer depth appears more than twice the main peak (depth value, 111) is a repeating sequence. The kmer depth appears at the half of the main peak (depth value, 27.5) means that this sequence is heterozygous. The total number of kmer obtained from the sequencing data was 41,072,179,362. After removing the kmer with abnormal depth, a total of 39,711,658,265 kmer were used for the genome size estimation, and the calculated genome length was about 710.83 Mbp, which was consistent with 654.40 Mbp estimated by flow cytometry [6]. According to the kmer distribution, the estimated repeat sequence ratio is about 54.56%. There is no obvious heterozygous peak, and the heterozygosity is a low value of 0.48%. In summary, the loquat had a simple genome, which is conducive to the assembly of the genome.

Nanopore, Hi-C and RNA Sequencing

Genomic DNA was extracted and sequenced following the Ligation Sequencing Kit (Nanopore, UK). The DNA was purified, and its quality was assessed by the Qubit 2.0 Fluorometer (Thermo Fisher, USA). The DNA was randomly interrupted and the fragments of ~20 kb were enriched and purified. Damaged DNA and ends were enzymatically repaired by NEBNext End Repair/dA-tailed

(NEB, UK). Then, a 20-kb library was constructed and sequenced by the Nanopore PromethION platform, according to the manufacturer's protocols. The data of about 106.23 Gb was obtained. After the data quality control, the final data volume was 100.10 Gb (Table 1). A Hi-C sample library was constructed by the fresh leaf of the loquat. The main process includes cross-linking DNA, restriction enzyme digestion, ends repair, DNA cyclization, and DNA purification. The library was sequenced by Illumina HiSeq 4000. A total of 67.25 Gb Clean Data was obtained and Q30 was 94.38%. RNA-seq samples were obtained by mixing an equal amount of RNA extracted from each tissue (leaf, fruit, bud, root, and branch) and used for library construction. After sequencing on the Illumina HiSeq 4000 platform, we obtained 11.06 Gb of sequencing data (Table 1).

Genome assembly based on Nanopore and Hi-C data

In Nanopore sequencing data, the N50 and average length of the reads reached 18.06 and 16.15 Kb respectively (Additional Table S1). According to the estimated genome size (710.83 Mbp), the sequencing depth was 131.69 x. The data were corrected by Canu software (Canu, RRID: SCR_015880, v1.4) [7] to obtain high-accuracy data for smartdenovo assembly, and then Racon software [8] and Pilon software (Pilon, RRID: SCR_01 4731, v1.21) [9] were used to calibrate the data. The total length of the draft genome sequence was 760.10 Mb composed of 597 contigs, and the contig N50 was 5.02 Mb.

BWA software (BWA, RRID: SCR_010910, v0.7.15) [10] was used to map the Hi-C short reads obtained from the Illumina HiSeq with the draft genome. The number of unique mapped Read pairs was 135,734,826, which accounted for 60.42 % of total Read pairs. These unique Read pairs were evaluated by HiC-Pro [11] to compare the valid interaction pairs and the invalid interaction pairs mapped to the draft genome. The result showed that the percent of valid interaction pairs was 73.97%. In conclusion, the Hi-C library has high quality. The contigs were interrupted by a length of 50 Kb and reassembled by Hi-C data. The position that could not be restored to the original assembled sequence was listed as a candidate error region, and then the low Hi-C coverage depth in this region confirmed this error. After the correction, 819 contigs were identified. LACHESIS software [12] was used to group, sort, and orient all contigs. 800 contigs could be mapped to 17 chromosomes. In the assembled process, 305 contigs were capable of determining the order and direction accounted for 676.24 Mb (89.27%), which were assembled to the chromosomes (Additional Table S2). Finally, 17 chromosomes and 514 unplaced scaffolds were obtained in the

final chromosome-level genome (Table 2). The scaffold N50 was 39.7 Mb.

Evaluation of assembly quality

The integrity of the assembled genome was assessed. Firstly, BWA software (BWA, RRID: SCR_010910, v0.7.15) [10] was used to compare the short reads obtained from the Illumina HiSeq sequencing data with the reference genome. The percent of mapped reads to the reference genome was up to 99.69%. Secondly, CEGMA (v2.5) [13] was used to assess the integrity of 458 conserved core genes for eukaryotes, 451 (98.47%) genes were present in the assembled genome. Thirdly, the database in Benchmarking Universal Single-Copy Orthologs (BUSCO, RRID:SCR_015008, v2.0) [14] was used to assess the completeness of gene regions, which contains 1,440 conserved core genes. The results showed that 96.81% of the plant single-copy orthologues were complete. Complete single-copy and complete multi-copy genes accounted for 64.65% and 32.15%, respectively. Therefore, these results indicated that the loquat genome assembly has high quality and coverage.

Genome annotation

LTR_FINDER (LTR_FINDER, RRID:SCR_015247) [15] and RepeatScout (RepeatScout, RRID:SCR_014653) [16] software were used to de novo predict repetitive sequences in the loquat genome, and then all isolated sequences were classified by PASTEClassifier [17] and mapped to the database of Repbase using RepeatMasker (RepeatMasker, RRID:SCR_012954) [18]. A total of 449.72 Mb of repeat sequences were identified, accounting for 59.17% of the genome size (Table S3). Among these repeat sequences, 48.6% (369.44 Mb) and 9.65% (73.34 Mb) were predicted as Class I transposons and Class II retrotransposons (Additional Table S3). In Class I, *Copia*- and Gypsy- retrotransposons account for 15.84% (120.38 Mb) and 26.28% (199.73 Mb) respectively. In Class II, TIR- and Helitron- transposons account for 6.85% and 1.96% respectively. The result showed that the retrotransposons account for a large proportion of the loquat genome.

The protein coding genes were predicted based on three different strategies, including de novo prediction, homologous species prediction, and Unigene prediction. Genscan (Genscan, RRID:SCR_012902) [19], Augustus (Augustus, RRID:SCR_015981, v2.4) [20], GlimmerHMM (GlimmerHMM, RRID:SCR_002654, v3.0.4) [21], GeneID (GeneID, RRID:SCR_002473, v1.4) [22], and SNAP (SNAP, RRID:SCR_005501) [23] were used in de novo prediction. GeMoMa (v1.3.1) [24] was used for prediction based on homologous species. The transcripts was assembled

by using Hisat (Hisat, RRID:SCR_015530, v2.0.4) [25] and Stringtie (v1.2.3) [26] based on RNA-seq data, and then GeneMarkS-T (v5.1) [27] and PASA (PASA, RRID:SCR_014656, v2.0.2) [28] were used for gene prediction. Finally, EVM (v1.1.1) [29] was used to integrate the prediction results obtained by the above three methods. The Venn diagram showed that 27,685 genes were predicted in all three strategies (Additional Fig. S1), and 45,743 genes accounted for 160.87 Mb were predicted (Additional Table S4). To better understand gene function, we searched all 45,743 protein-coding genes to protein databases, including InterProScan, KEGG, SwissProt, and TrEMBL. Results showed that 98.69% of the genes could be annotated from these databases. The distribution of repetitive sequences and protein coding genes were shown in Fig. 3B, 3C.

Based on the Rfam database [30], Blastn (Blastn, RRID:SCR_001598) was used for genome-wide alignment to identify microRNAs and rRNAs. tRNA was predicted by tRNAscan-SE (tRNAscan-SE, RRID:SCR:010835) [31]. A total of 656 tRNAs, 6,211 rRNAs, 121 miRNAs were predicted. GeneWise (GeneWise, RRID:SCR_015054) [32] was used to find immature stop codons and frameshift mutations in the predicted genes to obtain pseudogenes, and 7,642 pseudogenes were obtained.

Gene clusters and duplication

The protein sequence of *E. japonica* and its related six species (*Malus domestica*, *Prunus persica*, *Pyrus communis*, *Rubus occidentalis*, *Rosa chinensis*, and *Fragaria vesca*) were compared to analyze the duplication of genes and the classification of species-specific genes between species. The genome of all related species were downloaded from the database of Genome Database for Rosaceae. OrthoMCL software (OrthoMCL, RRID:SCR_007839) [33] was used to find the gene family unique to all species. In *E. japonica*, 45,743 genes were grouped into 17,333 gene families (Table 4), which was more than other species. The number of genes and gene families in *E. japonica* was similar with *P. communis*, which had 45217 genes and 16875 gene families. *E. japonica* had 665 unifamiles, suggesting these families were special in the loquat genome. The classification of genes showed that the single copy gene in loquat was less than other species, and 1849 single copy genes were identified. The loquat and pear had a large number of multiple copy genes (Fig. 4A). The gene expansion analysis showed that 182 genes were expanded in *E. japonica* compared with *M. domestica* and *P. communis*, including NB-ARC domain, transposase family tnp2, and Myb/SANT-like DNA-binding domain (Additional Table S5).

Due to limited computing power, fifty-one copy genes in loquat and six related species were randomly selected to construct a phylogenetic tree using MEGA (MEGA, RRID:SCR 000667, v7.0.26) software. The method of maximum-likelihood–based phylogenetic analyses were performed with *Rubus occidentalis* as the outgroup. Results indicated that the *Eriobotrya* has a close relationship with the *Malus* and *Pyrus* (Fig. 4). To further investigate the divergence time of these species, the MCMCTREE model was used. Fossil records were downloaded from the TIMETREE website [45] and used to calibrate the results. The divergence time of *Malus* and *Prunus* was set to 45.50 million years ago. Results showed that the loquat diverged from the *Malus* ∼6.76 million years ago (Fig. 4B).

4DTv (4-fold degenerate synonymous sites of the third codons) values were calculated according to the homologous gene pairs between the two species or the species itself. The 4DTv distribution map revealed two whole genome replication events. A divergence peak value (4DTv ∼ 0.01) was observed in the *E. japonica* -vs- *P. communis* in the map, and a low values were found in the *E. japonica* -vs- *R. chinensis* (Fig. 4C), which suggested that the divergence of *E .japonica* and *P. communis* was relatively later than the divergence of *E. japonica* and *R. chinensis*. In a self-alignment of the chromosomes based on gene synteny, a peak value (0.05) was found in 4DTv value, suggesting that a whole-genome or large-fragment duplication occurred in the *Eriobotrya* genome.

Chromosome evolution between the *Malus, Prunus*, and *Eriobotrya* genomes

The evolution of the *Eriobotrya* chromosomes and gene collinearity was evaluated using MCScan (version 0.8). The chromosomes of *Prunus* and *Malus* were used as reference genomes. A total of 26,557 and 40,928 gene pairs were found in inter-genomic comparison in *Eriobotrya* -vs- *Prunus* and *Eriobotrya* -vs- *Malus,* respectively. The alignments of syntenic chromosomes were visualized between *Malus*, *Prunus*, and *Eriobotrya* (Fig. 5A). The scattered points in *Eriobotrya* -vs- *Malus* were less than *Eriobotrya* -vs- *Prunus*, suggesting the close relationship between *Eriobotrya* and *Malus*. The frequency of large-scale fragment rearrangements among *Malus*, *Prunus*, and *Eriobotrya*, including inversions and translocations (Fig. 5B). In the comparison of *Prunus,* and *Eriobotrya*, Sac1, 4, and 8 in *Prunus* had duplicated (Fig. 5A). Sac1 divided into LG07/LG08 and LG06/LG15 in *Eriobotrya.* Sac4 and Sac8 were combined and formed LG01 and LG02. Sac5 was not duplicated and formed LG14 in *Eriobotrya*, suggesting that the other Sac5 was lost in the whole genome duplication. In the comparison of *Malus*, and *Eriobotrya*, C05 and C10 in *Malus* were

combined and formed LG01 and LG02 in *Eriobotrya*. C09 and C17 formed LG11 and LG13. One-to-one corresponding chromosome was not detected, suggesting that the fragment rearrangements widely occurred in the chromosomes of *Malus* and *Eriobotrya*. These findings implied that *Malus*, *Prunus*, and *Eriobotrya* shared some regions of chromosome and extensive chromosome rearrangements occurred. Overall, these findings a new insight on the evolution of *Eriobotrya* chromosomes.

Discussion

As far as our knowledge, this is the first report of the chromosome-level genome assembly of *E. japonica* using third-generation sequencing Nanopore and High-through chromosome conformation capture technology. A total of 45,743 high-quality protein coding genes were annotated by integrating results from 3 different methods including de novo prediction, homologous species prediction, and Unigene prediction. Phylogenetic analysis indicated that the *Eriobotrya* is closely related to the *Malus*. The analysis showed that a whole-genome or large-fragment duplication occurred in the *Eriobotrya* genome. The chromosomal rearrangement was found in *Eriobotrya* and *Malus*. This work provide a valuable chromosome-level genomic data for the loquat, and important genomic data for studying the loquat traits.

Abbreviations

4DTv: 4-fold degenerate synonymous sites of the third codons; BLAST: Basic Local Alignment Search Tool; bp: base pairs; Gb: gigabase pairs; GO: Gene Ontology; Hi-C: high-throughput chromosome conformation capture; HiSeq: high-throughput sequencing; kb: kilobase pairs; KEGG: Kyoto Encyclopedia of Genes and Genomes; Mb: megabase pairs; miRNA: MicroRNA; RNA-seq: RNA-sequencing; rRNA: ribosomal RNA; TrEMBL: a database of translated proteins from European Bioinformatics Institute; tRNA: Transfer RNA.

Conflict of interest

The authors declare that they have no competing interests.

Author contributions

Shuang Jiang performed the experiments and wrote the manuscript. Haishan An helped to collect the samples and revise the manuscript. Fangjie Xu helped to analyze the data and revise the manuscript. Xueying Zhang involved in designing the research and revised the manuscript. All authors read and approved the manuscript.

Availability of supporting data and materials

All sequencing data have been deposited in the BIG Data Center, Beijing Institute of Genomics (BIG), Chinese Academy of Sciences under BioProject number PRJCA001836. For genome assembly data, the accession number is GWHAAZU00000000 in Genome Warehouse in BIG. The run of clean reads in Nanopore, Illumina HiSeq, HiC, and RNA-seq were deposited in Genome Sequence Archive in BIG under the accession number of CRR078404~CRR078407. The annotation files of the genome (gff3, NR annotation files, et al.) was deposited in Baidu Cloud Disk (https://pan.baidu.com/s/1ALo86W-45mgvZ7MgrWLWvA, password: 1ku4).

Funding

This work was financed by a Grant from the National Natural Science Foundation of China (No. 31701886) and A Grant for Agriculture Applied Technology Development Program from Shanghai Agriculture Committee (2015-6-2-2).

References:

1. Lindley J. *Eriobotrya japonica*. Transactions of the Linnean Society of London. 1821;13 1:102.
2. Yang X, Glakpe K, Lin S, Hu Y, He Y, Nguyen TCN, et al. Taxa of plants of genus Eriobotrya around the world and native to Southeastern Asia. Joumal of Fruit Science (in Chinese). 2005;22 1:55-9.
3. Li GF, Zhang ZK and Lin SQ. Origin and evolution of eriobotrya. Acta Horticulturae. 2011;887:33-7.
4. Lo EY and Donoghue MJ. Expanded phylogenetic and dating analyses of the apples and their relatives (Pyreae, Rosaceae). Molecular phylogenetics and evolution. 2012;63 2:230-43. doi:10.1016/j.ympev.2011.10.005.
5. Doyle JJ and Doyle JL. A rapid DNA isolation procedure for small quantities of fresh leaf tissue. Phytochem Bull. 1987;19:11-5.
6. Zhang ZK, Wang YQ, Lin SQ and Du K. Ploidy identification of loquats for genome sequencing project by flow cytometry. Journal of Fruit Science. 2012;29 3:498-504 (in chinese).
7. Koren S, Walenz BP, Berlin K, Miller JR, Bergman NH and Phillippy AM. Canu: scalable and

accurate long-read assembly via adaptive k-mer weighting and repeat separation. Genome Res. 2017;27 5:722-36. doi:10.1101/gr.215087.116.

8. Vaser R, Sovic I, Nagarajan N and Sikic M. Fast and accurate de novo genome assembly from long uncorrected reads. Genome Res. 2017;27 5:737-46. doi:10.1101/gr.214270.116.

9. Walker BJ, Abeel T, Shea T, Priest M, Abouelliel A, Sakthikumar S, et al. Pilon: an integrated tool for comprehensive microbial variant detection and genome assembly improvement. Plos One. 2014;9 11:e112963. doi:10.1371/journal.pone.0112963.

10. Li H and Durbin R. Fast and accurate short read alignment with Burrows-Wheeler transform. Bioinformatics. 2009;25 14:1754-60. doi:10.1093/bioinformatics/btp324.

11. Servant N, Varoquaux N, Lajoie BR, Viara E, Chen CJ, Vert JP, et al. HiC-Pro: an optimized and flexible pipeline for Hi-C data processing. Genome Biol. 2015;16:259. doi:10.1186/s13059-015-0831-x.

12. Burton JN, Adey A, Patwardhan RP, Qiu R, Kitzman JO and Shendure J. Chromosome-scale scaffolding of de novo genome assemblies based on chromatin interactions. Nat Biotechnol. 2013;31 12:1119-25. doi:10.1038/nbt.2727.

13. Parra G, Bradnam K and Korf I. CEGMA: a pipeline to accurately annotate core genes in eukaryotic genomes. Bioinformatics. 2007;23 9:1061-7. doi:10.1093/bioinformatics/btm071.

14. Simao FA, Waterhouse RM, Ioannidis P, Kriventseva EV and Zdobnov EM. BUSCO: assessing genome assembly and annotation completeness with single-copy orthologs. Bioinformatics. 2015;31 19:3210-2. doi:10.1093/bioinformatics/btv351.

15. Xu Z and Wang H. LTR_FINDER: an efficient tool for the prediction of full-length LTR retrotransposons. Nucleic Acids Res. 2007;35 Web Server issue:W265-8. doi:10.1093/nar/gkm286.

16. Price AL, Jones NC and Pevzner PA. De novo identification of repeat families in large genomes. Bioinformatics. 2005;21 Suppl 1:i351-8. doi:10.1093/bioinformatics/bti1018.

17. Hoede C, Arnoux S, Moisset M, Chaumier T, Inizan O, Jamilloux V, et al. PASTEC: an automatic transposable element classification tool. Plos One. 2014;9 5:e91929. doi:10.1371/journal.pone.0091929.

18. Tarailo- Graovac M and Chen N. Using RepeatMasker to identify repetitive elements in genomic sequences. Current Protocols in Bioinformatics. 2009;4 10:11-4.

19. Burge C and Karlin S. Prediction of complete gene structures in human genomic DNA. Journal of molecular biology. 1997;268 1:78-94. doi:10.1006/jmbi.1997.0951.

20. Stanke M and Waack S. Gene prediction with a hidden Markov model and a new intron submodel. Bioinformatics. 2003;19 Suppl 2:ii215-25. doi:10.1093/bioinformatics/btg1080.

21. Majoros WH, Pertea M and Salzberg SL. TigrScan and GlimmerHMM: two open source ab initio eukaryotic gene-finders. Bioinformatics. 2004;20 16:2878-9. doi:10.1093/bioinformatics/bth315.

22. Blanco E, Parra G and Guigó R. Using geneid to identify genes. Current protocols in bioinformatics. 2007;4 3:1-28.

23. Korf I. Gene finding in novel genomes. BMC Bioinformatics. 2004;5:59. doi:10.1186/1471-2105-5-59.

24. Keilwagen J, Hartung F, Paulini M, Twardziok SO and Grau J. Combining RNA-seq data and homology-based gene prediction for plants, animals and fungi. BMC Bioinformatics. 2018;19 1:189. doi:10.1186/s12859-018-2203-5.

25. Kim D, Langmead B and Salzberg SL. HISAT: a fast spliced aligner with low memory requirements. Nature methods. 2015;12 4:357-60. doi:10.1038/nmeth.3317.

26. Pertea M, Pertea GM, Antonescu CM, Chang TC, Mendell JT and Salzberg SL. StringTie enables improved reconstruction of a transcriptome from RNA-seq reads. Nat Biotechnol. 2015;33 3:290-5. doi:10.1038/nbt.3122.

27. Tang S, Lomsadze A and Borodovsky M. Identification of protein coding regions in RNA transcripts. Nucleic Acids Res. 2015;43 12:e78. doi:10.1093/nar/gkv227.

28. Campbell MA, Haas BJ, Hamilton JP, Mount SM and Buell CR. Comprehensive analysis of alternative splicing in rice and comparative analyses with Arabidopsis. BMC Genomics. 2006;7:327. doi:10.1186/1471-2164-7-327.

29. Haas B, Salzberg S, Zhu W, Pertea M, Allen J, Orvis J, et al. Automated eukaryotic gene structure annotation using EVidenceModeler and the Program to Assemble Spliced Alignments. Genome Biol 2008;9:R7.

30. Griffiths-Jones S, Moxon S, Marshall M, Khanna A, Eddy SR and Bateman A. Rfam: annotating non-coding RNAs in complete genomes. Nucleic Acids Res. 2005;33 Database issue:D121-4. doi:10.1093/nar/gki081.

31. Lowe TM and Eddy SR. tRNAscan-SE: a program for improved detection of transfer RNA genes in genomic sequence. Nucleic Acids Res. 1997;25 5:955-64. doi:10.1093/nar/25.5.955.

32. Birney E, Clamp M and Durbin R. GeneWise and Genomewise. Genome Res. 2004;14 5:988-95. doi:10.1101/gr.1865504.

33. Li L, Stoeckert CJ, Jr. and Roos DS. OrthoMCL: identification of ortholog groups for eukaryotic genomes. Genome Res. 2003;13 9:2178-89. doi:10.1101/gr.1224503.

Figure legends

Figure 1 The loquat of seventh star (*Eriobotrya japonica*).

Figure 2 The Kmer analysis (K=23) of *Eriobotrya japonica* genome characteristics.

Figure 3 Summary of the de novo genome assembly and sequencing analysis of *Eriobotrya japonica*. A, Chromosome number; B, numbers of repeat sequences per Mb; C, numbers of protein coding genes per Mb; and D paralogous relationships between E. *japonica* chromosomes.

Figure 4 The genome evolution of *Eriobotrya*. (A) Comparison of copy numbers in gene clusters of *Eriobotrya* genomes and six related species genomes. (B) Constructed phylogenetic tree and divergence time estimation. (C) 4DTv analyses in *Eriobotrya* and related species.

Figure 5 The chromosomes collinearity among *Malus*, *Prunus* and *Eriobotrya*. (A) The inter-genomic comparison. (B) The chromosomes map in three species.

Additional files

Additional Figure S1 COG function classification of all unigenes.

Additional Table S1 The sequence length of reads in Nanopore.

Additional Table S2 The details of the distribution of each chromosome sequences

Additional Table S3 The details of repeat sequences in the loquat genome.

Additional Table S4 Gene prediction result statistics

Additional Table S5 The number of expansion gene in E. japonica compared with *M. domestica* and *P. communis*.

Table 1: Sequencing data used for loquat genome assembly and annotation

Sequencing type	Platform	Library size (bp)	Clean data (Gb)	Application
Genome short reads	Illumina HiSeq 4000	350	47.42	Genome survey and assessment
Nanopore reads	Nanopore platform	20000	100.10	Contig assembly
Hi-C reads	Illumina HiSeq 4000	300-700	67.25	Chromosome construction
Transcriptome short reads	Illumina HiSeq 4000	200-500	11.06	Genome annotation and assessment

Table 2 Assembly statistics

	Software	Assembly level	Number of sequences	N50 (Mb)	size (Gb)
Nanopore	Smartdenovo, Racon, and Pilon	contig	597	5.0	760.1
Nanopore and Hic	Lachesis	chromosome	17 + 514[a]	39.7	676.2 + 83.9

[a]There are 514 unplaced scaffolds in the final chromosome-level assembly. These unplaced contigs comprise ~10.73% of total bases in the genome assembly size.

Table 3 Repeat sequences in the loquat genome

Type*	Number	Length (bp)	Rate (%)
ClassI/LTR/Copia	141,908	120,380,193	15.84
ClassI/LTR/Gypsy	183,863	199,727,884	26.28
ClassII/Helitron	45,852	14,912,320	1.96
ClassII/TIR	140,384	52,101,491	6.85
Other	184,400	62,606,412	8.24
Total	669,919	449,728,153	59.17

*The main type of repeat sequences were shown.

Table 4 The statistics of gene family classification in seven species.

Species	Total genes	Cluster number	Total family	Uni family
E. japonica	45,743	39,294	17,333	665
M. domestica	28,306	20,426	12,797	365
P. communis	45,217	32,764	16,875	819
P. persica	26,873	22,583	14,969	310
R. occidentalis	33,253	24,641	15,479	1,241
F. vesca	24,034	21,789	14,859	196
R. chinensis	30,214	26,705	15,326	473

Figure 1

Click here to download Figure Fig 1.tif

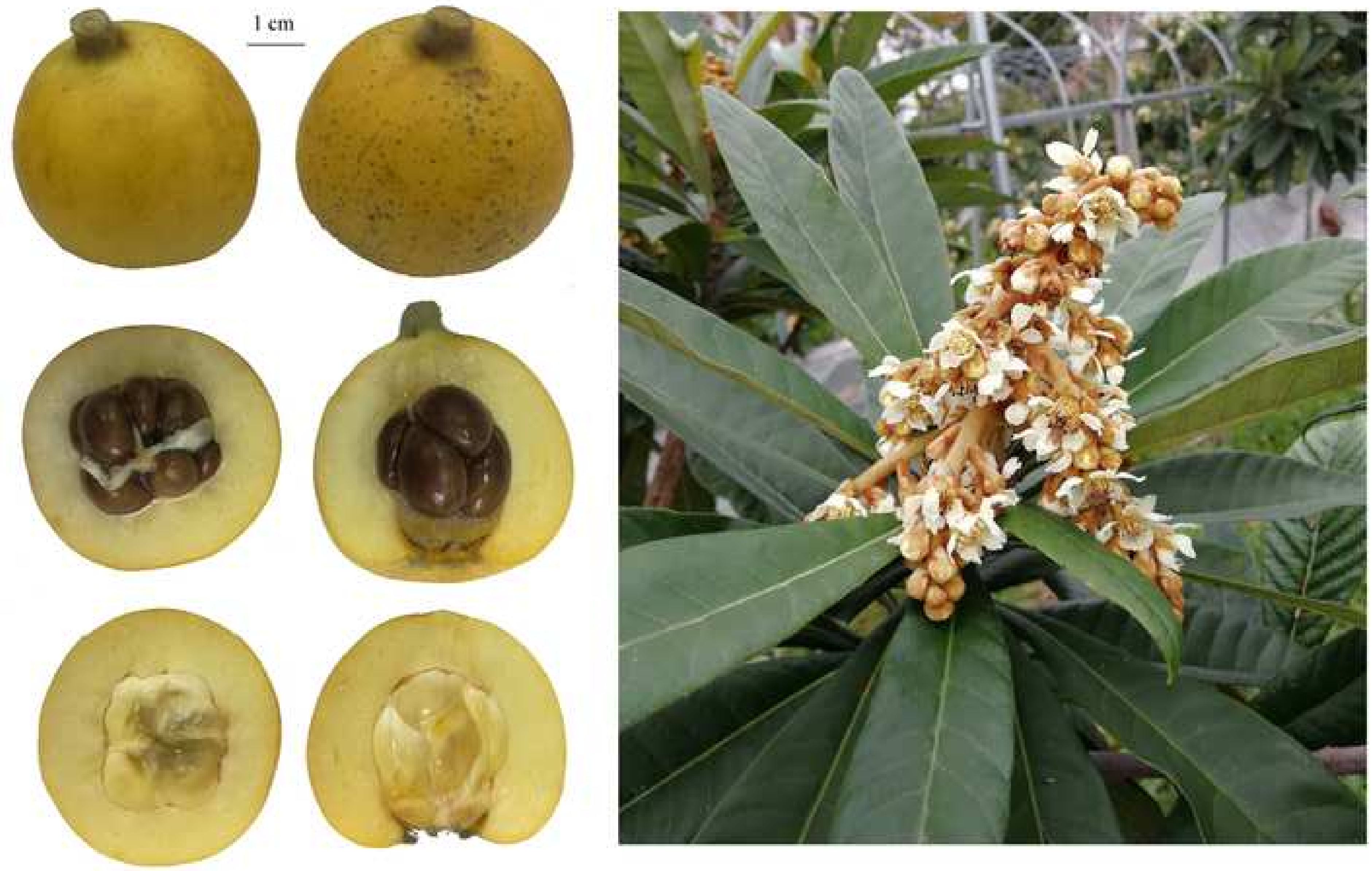

Figure 2

Click here to download Figure Fig 2.png

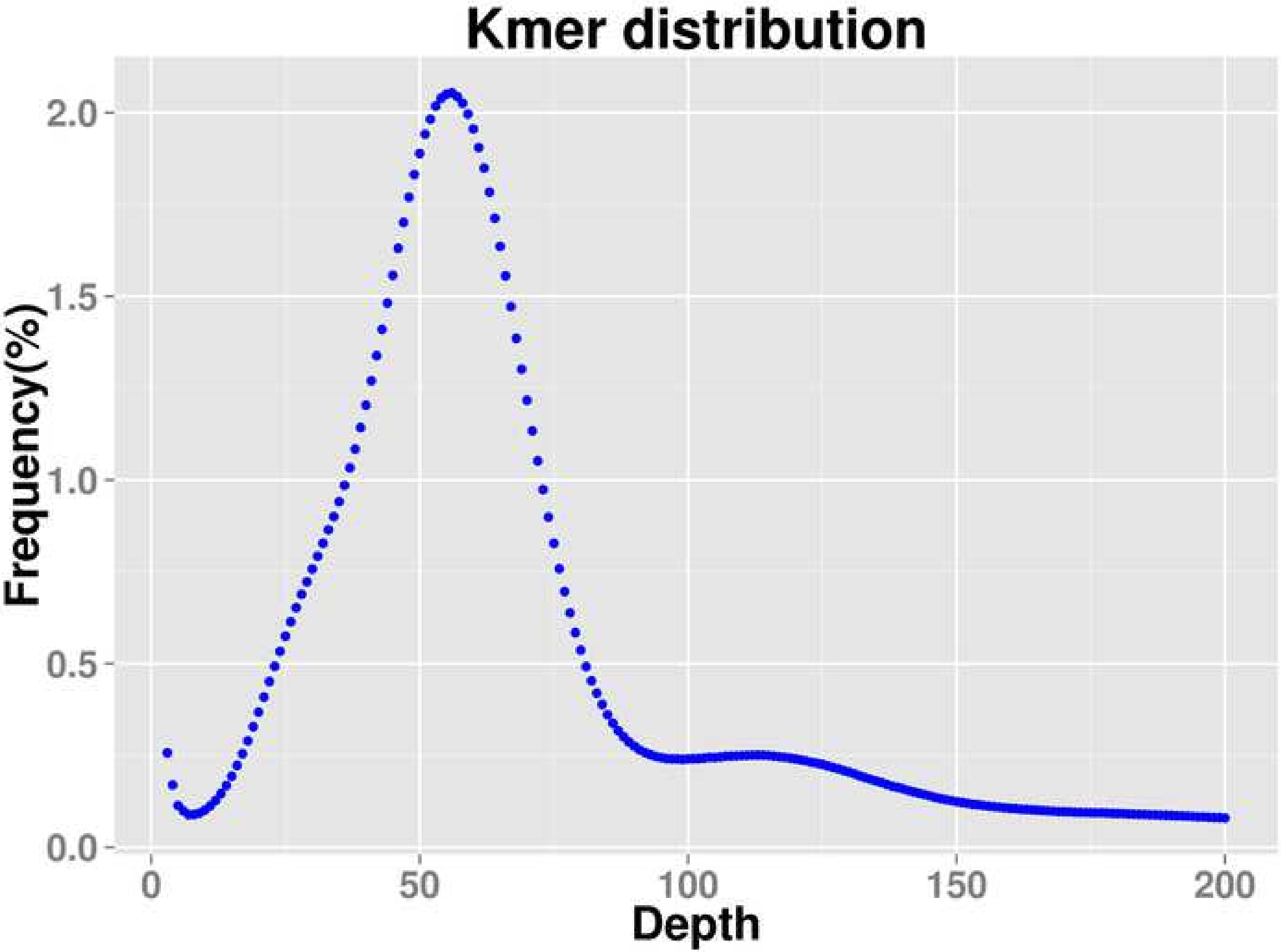

Figure 3

Click here to download Figure Fig 3.png

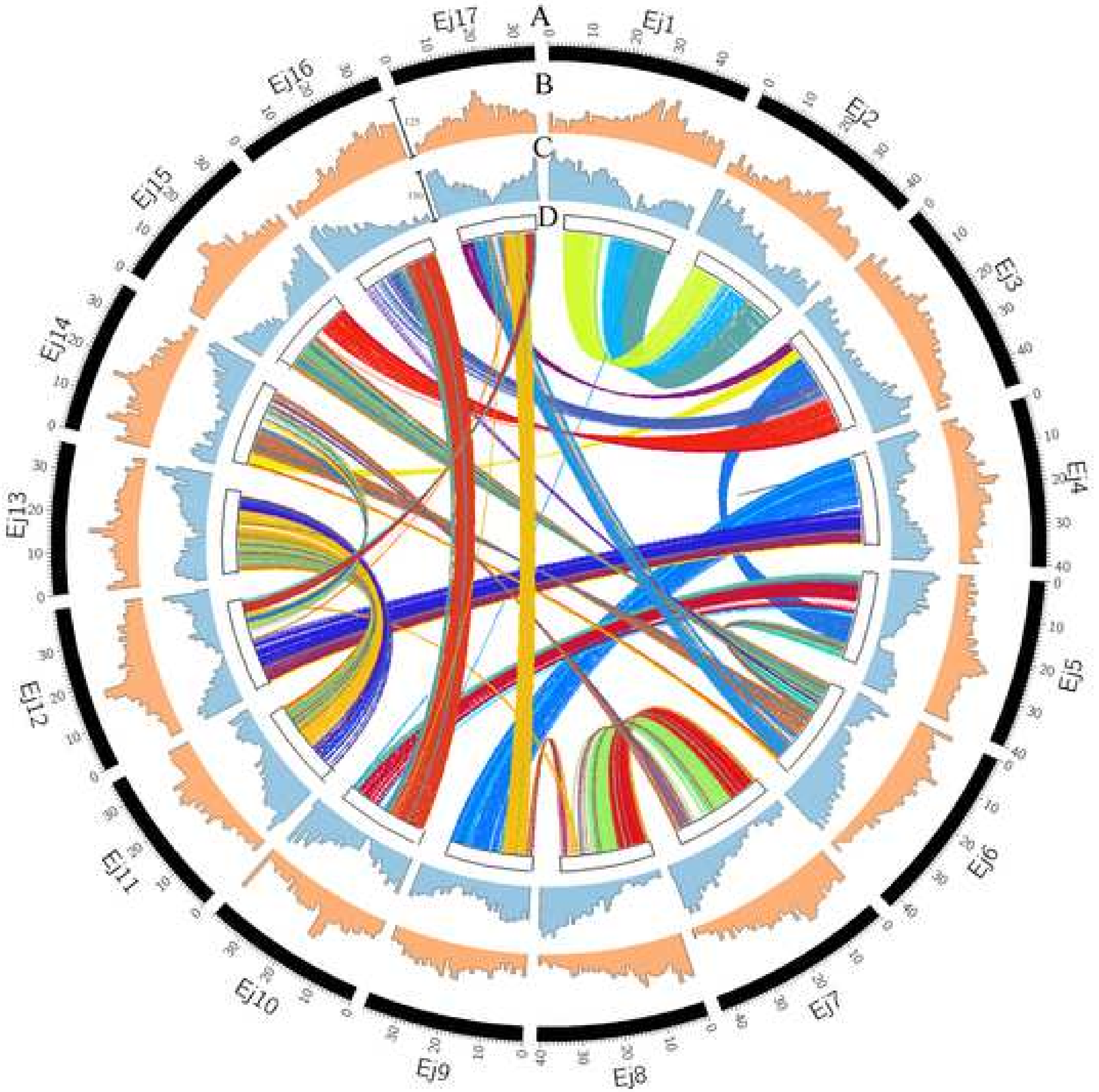

Figure 4 Click here to download Figure Fig 4.tif

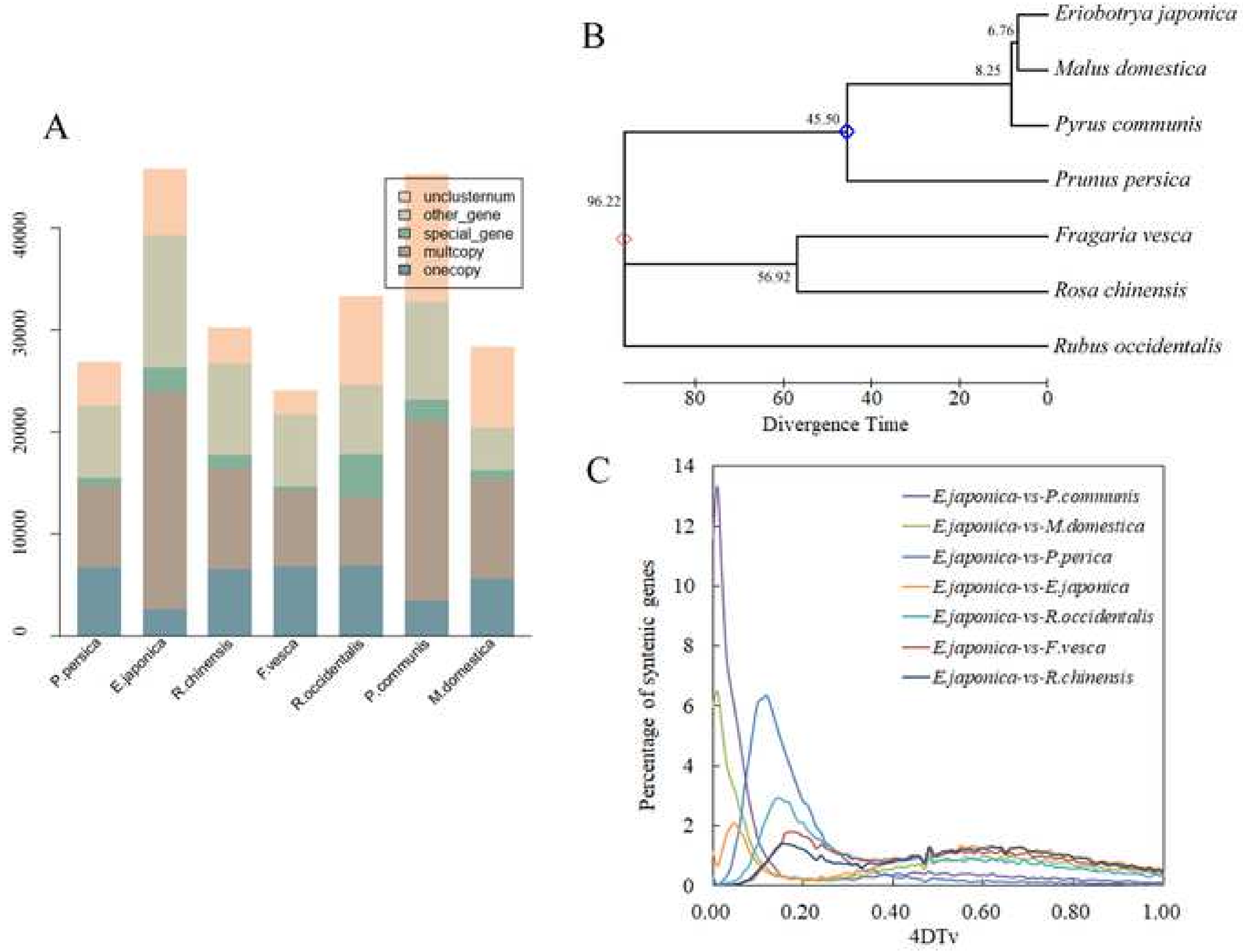

Figure 5

Click here to download Figure Fig 5.tiff

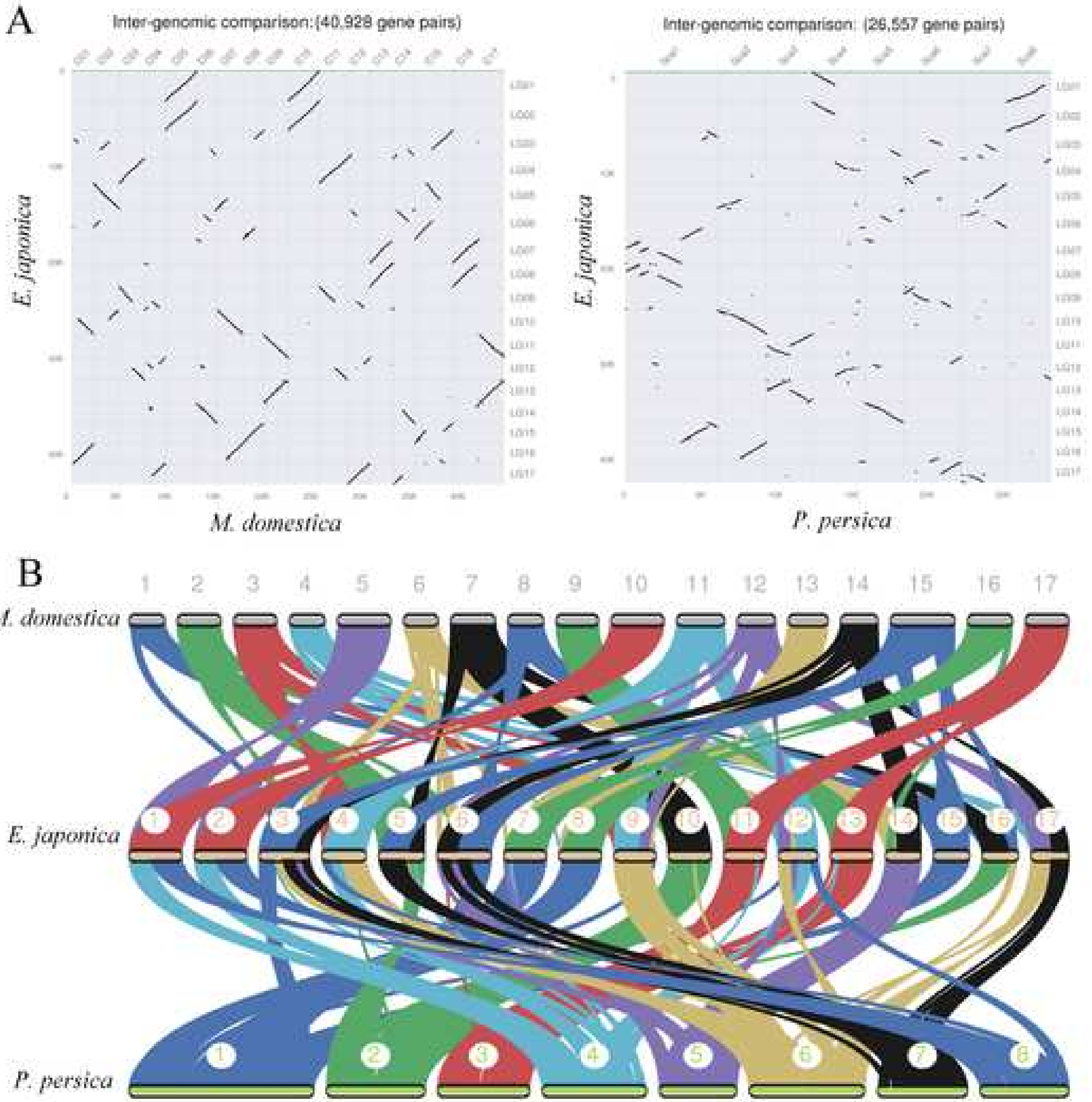

Additional Figure S1

Click here to access/download
Supplementary Material
Fig S1.png

Additional Table S1

Click here to access/download
Supplementary Material
Table S1.xlsx

Additional Table S2

Click here to access/download
Supplementary Material
Table S2.xlsx

Additional Table S3

Click here to access/download
Supplementary Material
Table S3.xlsx

Additional Table S4

Click here to access/download
Supplementary Material
Table S4.xlsx

Additional Table S5

Click here to access/download
Supplementary Material
Table S5.xlsx
