# Supplementary material for: Chromosome-level genome assembly and annotation of the loquat (Eriobotrya japonica) genome
Source: Gigascience. 2020 Mar 6;9(3):giaa015. doi: 10.1093/gigascience/giaa015 (PMC7059265; doi:10.1093/gigascience/giaa015)
Supplement: giaa015_GIGA-D-19-00365_Revision_1 [file giaa015_giga-d-19-00365_revision_1.pdf]

GigaScience

Chromosome-level genome assembly and annotation of the loquat (Eriobotrya japonica) genome

--Manuscript Draft--

**Manuscript Number:**	GIGA-D-19-00365R1
**Full Title:**	Chromosome-level genome assembly and annotation of the loquat (Eriobotrya japonica) genome
**Article Type:**	Data Note
**Funding Information:**	a Grant for Agriculture Applied Technology Development Program from Shanghai Agriculture Committee (2015-6-2-2) — Dr. Xueying Zhang<br>National Natural Science Foundation of China (31701886) — Dr. Shuang Jiang
**Abstract:**	Background<br><br>The loquat (Eriobotrya japonica) is a species of flowering plant in the family Rosaceae that is widely cultivated in Asian, European, and African countries. It blossoms in the winter and ripens in the early summer. The genome of loquat has not been reported, which limits the study of molecular biology in the loquat. Here, we used the third-generation sequencing technology of Nanopore and High-through chromosome conformation capture (Hi-C) technology to sequence the genome of Eriobotrya to provide a reference for researchers.<br><br>Findings<br><br>We generated 100.10 Gb of long reads using Nanopore sequencing technologies. Three types of Illumina high-throughput sequencing data, including Genome short reads (47.42 Gb), transcriptome short reads (11.06 Gb) and Hi-C short reads (67.25 Gb), were also generated to construct the loquat genome. All data were assembled into a 760.1 Mb genome assembly. The contigs were mapped to chromosomes by using Hi-C technology based on the contacts between contigs, and then assembled a genome exhibiting 17 chromosomes and a scaffold N50 length of 39.7 Mb. A total of 45,743 protein-coding genes were annotated in the Eriobotrya genome, and we investigated the phylogenetic relationships between the Eriobotrya and six other Rosaceae species. Eriobotrya shows a close relationship with Malus and Pyrus , and the divergence time of Eriobotrya and Malus was 6.76 million years ago. Furthermore, chromosome rearrangement was found in Eriobotrya and Malus .<br>Conclusions: We constructed the first high-quality chromosome-level Eriobotrya genome using Illumina, Nanopore, and Hi-C technologies. This work provides a valuable reference genome for molecular studies of the loquat and provides new insight into chromosome evolution in this species.
**Corresponding Author:**	Xueying Zhang<br><br>CHINA
**Corresponding Author Secondary Information:**	
**Corresponding Author's Institution:**	
**Corresponding Author's Secondary Institution:**	
**First Author:**	Shuang Jiang
**First Author Secondary Information:**	
**Order of Authors:**	Shuang Jiang<br>Haishan An

Powered by Editorial Manager® and ProduXion Manager® from Aries Systems Corporation

	Fangjie Xu
	Xueying Zhang
**Order of Authors Secondary Information:**	
**Response to Reviewers:**	Dear editor:<br>Thank you very much for your letter. We greatly appreciate the suggestions and comments made by you and the reviewers. Based upon the suggestions and comments, we have revised the manuscript and our manuscript has been edited by AJE English Editing. Specific revisions made on the manuscript are shown below as well as in the new manuscript in red. We hope that the manuscript is now suitable for publication in GigaScience.<br><br>Sincerely yours,<br>Xueying Zhang<br><br>The corrections and responses to editor and reviewers' comments and suggestions are as follows:<br><br>Editor comments:<br>Please register any new software application in the bio.tools and SciCrunch.org databases to receive RRID (Research Resource Identification Initiative ID) and biotoolsID identifiers, and include these in your manuscript.<br><br>Done. All new software applications have a RRID, and we registered them in bio.tools.<br><br><br>Reviewer reports:<br>Reviewer #1: Jiang et. al report a chromosome scale genome assembly of the important horticultural crop loquat. They utilized a single-molecule, Nanopore sequencing based approach coupled with Hi-C to generate a high-quality assembly. Loquat and apple have a clear2:2 synteny with a high degree of collinearity, suggesting they have a shared whole genome duplication event and that the loquat genome is high-quality. The resources presented here will be useful for the loquat and Rosaceae research communities as well as the comparative genomics communities. I have a few comments/suggestions that I feel with strengthen the manuscript.<br><br>Thank you for your comments. We really appreciate your suggestion.<br><br>Major:<br>1. Significantly more details are needed for the genome assembly section. Based on the methods, it seems like Canu was used to error correct the reads and smartdenovo assembly was used to assemble them into contigs. Then, Racon and Pilon were used to polish the assembly. This is an unusual pipeline to use and it is unclear which data was used in each step. Was the full Canu pipeline used to assemble a draft genome prior to smartdenovo assembly? Or, were error corrected or corrected and trimmed reads used as input for smartdenovo? Pilon requires Illumina short reads for polishing, was the HiSeq4000 data used for this? How many rounds of Racon and Pilon were run on the data? Statistics for each step of the assembly would also be helpful (e.g. how many errors were corrected, the input metrics for smartdenovo, etc.).<br><br>Thank you for your comments. A full Canu run includes three stages: correction, trimming, and assembly. We tested the full Canu pipeline to assemble a draft genome. The result showed that the genome size was 280,096,430bp with N50 85,570bp, which was not a good result. We changed our strategy. Canu was only used to correct the Reads by the stage of correction, and then the corrected Reads (genome.correctedReads.fasta) were assembled by SMARTdenovo to obtain the draft genome. The assembly method of Canu+SMARTdenovo was also reported in other studies (e.g. Schmidt MH , Vogel A , Denton AK , et al. 2017. De novo assembly of a new Solanum pennellii accession using Nanopore Sequencing. The Plant Cell, tpc.00521.). Racon used the Nanopore Reads and Pilon used the genome short reads from Illumina HiSeq 4000. The errors were not recorded in Canu and Racon. The error radio of 1.64%, 0.07%, and 0.01% were recorded in 3 rounds in Pilon. We revised this paragraph as "First, the Nanopore Reads were corrected by the correction function in

Powered by Editorial Manager® and ProduXion Manager® from Aries Systems Corporation

Canu (Canu, RRID:SCR_015880, v1.4) [8]. Second, the corrected reads (6,198,187 reads) were assembled by SMARTdenovo (SMARTdenovo, RRID:SCR_017622) [9] to obtain the draft genome with 597 contigs covering 732.25 MB. Third, Racon (Racon, RRID:SCR_017642) [10] was used to calibrate the draft genome with Nanopore reads through three rounds, and the genome size was corrected to 753.38 Mb. Fourth, Pilon (Pilon, RRID: SCR_014731, v1.21) [11] was used to calibrate the draft genome with short genome reads from the Illumina HiSeq 4000 platform through 3 rounds with error radio of 1.64%, 0.07%, and 0.01%, respectively. Finally, the total length of the draft genome sequence was 760.10 Mb, composed of 597 contigs, and the contig N50 was 5.02 Mb."

2. The manuscript contains would benefit from heavy editing for clarity.

We really appreciate your suggestion.

Minor:

1. Line 65: "The reads were searched by the NT database, which confirmed that the sample is free from contamination." It is unclear what this means. My interpretation is that a subset of reads were queried against a database using BLAST or another alignment program to identify contaminant sequences. More details should be provided here.

We added a new sentence as "Ten thousand reads were randomly selected to search the NT database using BLAST, and 90.62% of the reads were mapped to the Malus and Pyrus genomes. No reads were mapped to microorganisms or animals, which confirmed that the sample was free from contamination."

2. Line 82. A heterozygosity rate of 0.48 may be low relative compared to other highly heterozygotic species, but it would likely still present a challenge for genome assembly. Smartdenovo assembly will smash haplotypes together but programs like Canu should keep them separate during assembly. Was the full Canu pipeline run on the assembly? If so, how does this compare to the Smartdenovo assembly?

Canu was only used to correct the Reads by the stage of correction. The corrected Reads were assembled by SMARTdenovo to obtain the draft genome.

3. Line 87 Interrupted to sheared

Done.

4. Line 92. A protocol should be referenced for the HiC library construction

We added a reference [7].

5. Line 106. Parameters should be reported for aligning the HiC reads to the genome using BWA.

The comparison mode was 'aln', and the other parameters were default. We added a sentence as "The comparison mode was 'aln', and the other parameters were set to the defaults." in the revised manuscript.

6. Line 111. Interrupted to split

Done.

7. Line 116. It is unclear how 800 contigs were mapped to 17 chromosomes, but only 305 were oriented into the 17 pseudomolecules.

819 contigs (305+495+19, 760.1 Mb) were identified. 305 contigs (676.24 Mb, 88.97%) were capable of determining the order and direction. 495 contigs (81.29 Mb, 10.69%) could be mapped to some chromosomes, but their order and direction were not clear. 19 contigs (2.57 Mb) were not mapped to some chromosomes. 305 contigs account for 88.97% in the whole genome. We added some details in the revised manuscript.

Powered by Editorial Manager® and ProduXion Manager® from Aries Systems Corporation

8. Line 148. More details should be provided on how transcripts were assembled and what cutoffs were used. Hisat and Stringtie are listed, but no details are provided.

In most software, we used the default parameters. The usage of Hisat and Stringtie was based on an added reference [29]. The prediction result in this paragraph was shown in Additional Table S3.

9. Line 196. Loquat and apple have clear 2:2 synteny and shared Ks peaks, but it is not explicitly mentioned that they share a common whole genome duplication event.

We added it in the manuscript as "Eriobotrya and Malus presented clear 2:2 synteny, implying that they shared a common whole-genome duplication event".

Reviewer #2: Jiang et al. reported the high quaility genome assembly and annotation for an important fruit tree, Eriobotrya japonica. In my opinion, this study is original, and data analysis have been well planned and conducted. The genomic resources and analysis are valuable for the loquat community and more broader regime of genomics and plant biology. However, there are large spaces for improvement in the English expression. I think editing by a native speaker is necessary.
It could be accepted after minor revision.

Thank you for your comments. We really appreciate your suggestion. Based upon the suggestions and comments, we have revised the manuscript and our manuscript has been edited by AJE English Editing.

Major concern:
Please provide parameters and settings for specific analysis you conducted, especially for the genome assembly part.

We revised this paragraph as "First, the Nanopore Reads were corrected by the correction function in Canu (Canu, RRID:SCR_015880, v1.4) [8]. Second, the corrected reads (6,198,187 reads) were assembled by SMARTdenovo (SMARTdenovo, RRID:SCR_017622) [9] to obtain the draft genome with 597 contigs covering 732.25 MB. Third, Racon (Racon, RRID:SCR_017642) [10] was used to calibrate the draft genome with Nanopore reads through three rounds, and the genome size was corrected to 753.38 Mb. Fourth, Pilon (Pilon, RRID: SCR_014731, v1.21) [11] was used to calibrate the draft genome with short genome reads from the Illumina HiSeq 4000 platform through 3 rounds with error radio of 1.64%, 0.07%, and 0.01%, respectively. Finally, the total length of the draft genome sequence was 760.10 Mb, composed of 597 contigs, and the contig N50 was 5.02 Mb."

Minor comments:
(I am not a native speaker. Here, I pick up specific comments related to generally the language expression)
1. line 11, "It flowered", is it a good expression?

We revised "flowered" as "blossoms".

2. line 19-21, please check this sentence, "The Hi-C ,,,, 39.7 Mb". Do you think Hi-C technology could really do assembly?

We revised this sentence as "The contigs were mapped to chromosomes by using Hi-C technology based on the contacts between contigs"

3. line 22, "analyzed" -> "investigated"

Done.

4. line 23, "the other six Rosaceae" -> "six other Rosaceae"

Done.

5. line 33, "are classified by" -> "were identified by"

Done.

6. line 34, "were" -> "are"

Done.

7. line 40, "The top buds become flowers", why do you want to say this?

We deleted this sentence.

8. line 41, why do you want to use "flowered"?

This word was revised as “blossoms”

9. line 66-67, "Evaluation of the chloroplast of the species ,,, content", why do you want to do that? How did you do that? Is it relevant?

It suggested that the nuclear DNA were sequenced, not chloroplast DNA. It is relevant, and we deleted this sentence.

10. line 82, hard to imagine a simple genome, please define it if you want to describe your assembly as a simple one.

Thank you for your comments. We deleted this sentence.

11. lines 91-92, "A Hi-C ,,, of the loquat", problematic expression. Do you really think the fresh leaf can do Hi-C? Please carefully check the full paper, for this similar problem.

We revised this sentence as “A Hi-C sample library was constructed from genomic DNA from the fresh leaves of the loquat”

12. line 94, "by" -> "with".

This sentence was revised as “The library was sequenced on the Illumina HiSeq 4000 platform.”

13. any reference for "smartdenovo"?

We added a reference [9].

14. line 116, "assembled" -> "assembly"

Done.

15. no need to use "software" so often.

We deleted "software".

16. line 173, please define "unifamiles". and any type there.

We revised "unifamiles" as “unique families”.

17. line 176, if you reported results of gene expansion. Please describe how you did do that?

We added a new sentence here as “CAFE (CAFE, RRID:SCR_005983) was used to study gene family expansion [38]”.

18. line 209-210, "One-to-one corresponding chromosome", hard to explain this. Please define it or make it clear.

Powered by Editorial Manager® and ProduXion Manager® from Aries Systems Corporation

It means corresponding one by one. We deleted this sentence.

19. line 216, "Discussion" -> "Conclusion"

Done.

20. line 258, please carefully check your reference list. A lot bugs.

Done

21. line 345, a new title would be "Picture of a loquat variety, seventh star"

Done.

22. Table 1 and 2, could be moved to the supplementary.

Table 1 and 2 showed some data. Can we keep it in the manuscript?

23. Replace Table 3 with Table S3.

Done.

24. line 384, please define "Uni family"

We revised "unifamiles" as “unique families”.

25. Figure 4, make it clear, by define the items you used or any other means.

Done. The figures in the pdf file were not original one, pictures lost clarity during conversion. The high quality original figures could be downloaded when you click the website in upper right corner in the picture page.

Reviewer #3: This paper is worth publishing for the Data Note for GigaScience because the authors have constructed a highly accurate genome and gene sequence of loquat. The method is reasonable and the presentation is pretty good. Speaking of greed, since it is clear that the relationship with Malus is relatively close among the fruit trees of the Rosaceae family, so there should be presented some discussion about the traces on the genome that triggered the differentiation of morphological features between both species, I recommend that as it would be done for the future work. The following minor concerns should be corrected before acceptance.

Thank you for your comments. We really appreciate your suggestion.

Minor concerns
1. L58 How to extract the RNA from the collected samples should be described.

We revised this sentences as “The leaves, fruit, buds, roots, and branches were collected for RNA extraction via the CTAB-LiCl method.”

2. L63 double-end would be pair-end.

Done.

3. L71 the appearance of the equation is not clear. Please correct the format of the equation.

We enlarged the font size.

4. L86 "quality" have to be "quantity", because the Qubit 2.0 Fluorometer is the device to evaluate the quantity of the DNA/RNA with fluorescence.

Done.

Powered by Editorial Manager® and ProduXion Manager® from Aries Systems Corporation

	5. L89 How many flow cells have been used with the PromethION platform to acquire about the 106.23 Gb.<br><br>Two flow cells have been used. We revised this sentence as “Then, a 20-kb library was constructed and sequenced on the Nanopore PromethION platform in two flow cells”.<br><br>6. L205 "Sac1, 4, and 8" has been suddenly appeared at the text. Please describe about relationships between the former and later text to be clearly understood, although chromosome scale duplication is very interesting.<br><br>We revise this sentence as “the Sac1, 4, and 8 chromosomes of Prunus were found to be duplicated”<br><br>7. L349 "de novo" should be written in italic.<br><br>Done.<br><br>8. L364 Add period.<br><br>Done.<br><br>9. L366 Add period.<br><br>Done<br><br>10. L367 E. japonica should be written in italic.<br><br>Done.<br><br>11. L375 "Hic" should be "Hi-C"<br><br>Done.<br><br>12. Figures Provide higher resolutional figures than current version. Because those are not clear. Figure 4A Correct the overlap of the legends on a bar. Figure 5A It is difficult to see the scale of the figure. Please provide higher resolutional figures.<br><br>We revised the Figure 4A. The figure in pdf file was not the original one. The high quality original figure could be downloaded when you click the website in upper right corner in the picture page.
**Additional Information:**	
**Question**	**Response**
Are you submitting this manuscript to a special series or article collection?	No
**Experimental design and statistics**<br><br>Full details of the experimental design and statistical methods used should be given in the Methods section, as detailed in our Minimum Standards Reporting Checklist. Information essential to interpreting the data presented should be made available in the figure legends.<br><br>Have you included all the information requested in your manuscript?	Yes

**Resources**<br><br>A description of all resources used, including antibodies, cell lines, animals and software tools, with enough information to allow them to be uniquely identified, should be included in the Methods section. Authors are strongly encouraged to cite Research Resource Identifiers (RRIDs) for antibodies, model organisms and tools, where possible.<br><br>Have you included the information requested as detailed in our Minimum Standards Reporting Checklist?	Yes
**Availability of data and materials**<br><br>All datasets and code on which the conclusions of the paper rely must be either included in your submission or deposited in publicly available repositories (where available and ethically appropriate), referencing such data using a unique identifier in the references and in the “Availability of Data and Materials” section of your manuscript.<br><br>Have you have met the above requirement as detailed in our Minimum Standards Reporting Checklist?	Yes

Powered by Editorial Manager® and ProduXion Manager® from Aries Systems Corporation

Manuscript

Click here to access/download;Manuscript;365-2019.12.19revised.docx

Chromosome-level genome assembly and annotation of the loquat (*Eriobotrya japonica*) genome

Shuang Jiang[†], Haishan An[†], Fangjie Xu, Xueying Zhang*

Forestry and Pomology Research Institute, Shanghai Key Lab of Protected Horticultural Technology, Shanghai Academy of Agricultural Sciences, Shanghai, 201403, China

[†]Equal contribution.

***Correspondence:** Xueying Zhang; loquat_zhang@126.com, Tel: 86 021 52355473

ORCIDs：

Shuang Jiang, 0000-0002-4675-7143

Haishan An, 0000-0001-6358-6529

Fangjie Xu, 0000-0001-8902-3986

Xueying Zhang, 0000-0002-9797-1131

Abstract

Background: The loquat (*Eriobotrya japonica*) is a species of flowering plant in the family Rosaceae that is widely cultivated in Asian, European, and African countries. It blossoms in the winter and ripens in the early summer. The genome of loquat has to date not been published, which limits the study of molecular biology in this cultivated species. Here, we used the third-generation sequencing technology of Nanopore and High-through chromosome conformation capture (Hi-C) technology to sequence the genome of *Eriobotrya* to provide a reference for researchers. **Findings:** We generated 100.10 Gb of long reads using Oxford Nanopore sequencing technologies. Three types of Illumina high-throughput sequencing data, including Genome short reads (47.42 Gb), transcriptome short reads (11.06 Gb) and Hi-C short reads (67.25 Gb), were also generated to help construct the loquat genome. All data were assembled into a 760.1 Mb genome assembly. The contigs were mapped to chromosomes by using Hi-C technology based on the contacts between contigs, and then assembled a genome exhibiting 17 chromosomes and a scaffold N50 length of 39.7 Mb. A total of 45,743 protein-coding genes were annotated in the *Eriobotrya* genome, and we investigated the phylogenetic relationships between the *Eriobotrya* and six other Rosaceae species. *Eriobotrya* shows a close relationship with *Malus* and *Pyrus*, with the divergence time of *Eriobotrya*

and *Malus* being 6.76 million years ago. Furthermore, chromosome rearrangement was found in *Eriobotrya* and *Malus*. **Conclusions:** We constructed the first high-quality chromosome-level *Eriobotrya* genome using Illumina, Nanopore, and Hi-C technologies. This work provides a valuable reference genome for molecular studies of the loquat and provides new insight into chromosome evolution in this species.

Data Description

Background

The genus *Eriobotrya* L. (common name loquat) is a species of flowering plant in the family Rosaceae [1], including approximately twenty-five species identified by most taxonomists. Sixteen of the species are native in China [2]. Cultivated loquats in Asia mainly belong to *Eriobotrya japonica* (NCBI: txid 32224). The loquat originated from China and has been produced widely throughout other Asian countries (Japan and Korea), some southern European countries (Turkey, Italy, and France), and several northern African countries (Morocco and Algeria) [3]. This species is a large evergreen tree that is grown commercially for its yellow or red fruit. The relationships of loquat, apple, pear, and peach are close [4]. In contrast, the maturity period of the loquat is early summer, which is earlier in the year than most of other cultivated fruits. The loquat is evergreen and blossoms in winter. After flower bud differentiation, the loquat blossoms without a long period of dormancy. The loquat exhibits infinite inflorescences, and one inflorescence produce many fruits, which increases the ability to adapt to the low temperature in the winter.

In the present study, we generated a genome assembly for the loquat with 17 chromosomes and a genome size of 760 Mb. The genome assembly was created using Nanopore long reads and Hi-C data. Illumina paired-end sequence was used for the base and indel correction. The completeness and continuity of the genome were comparable with those of other important Rosaceae species. The high-quality reference genome generated in this study will facilitate research on population genetic traits and functional gene identification related to important characteristics of the loquat.

Sample collection

Eriobotrya japonica cv. Seventh Star is a cultivar bred by the team of Dr. Xueying Zhang at the Shanghai Academy of Agricultural Sciences (SAAS, Shanghai, China) (Fig. 1) that is widely cultivated in Shanghai, China. Young leaves were collected from an individual of Seventh Star on

Mar. 20, 2019 at the experimental farm of SAAS in Zhuanghang Town (Fengxian, Shanghai, China). This tree was 14 years old and was considered to be in the adult phase. The leaves were frozen in liquid nitrogen and stored at −80 °C until DNA extraction. Total genomic DNA was extracted from the leaf tissues following the CTAB protocol [5]. The leaves, fruit, buds, roots, and branches were collected for RNA extraction via the CTAB-LiCl method.

Estimation of genome size and heterozygosity analysis

The qualified genomic DNA was randomly disrupted by ultrasonic oscillation to generate the fragments of 350 bp, and then a small fragment sequencing library was constructed by terminal repair, the addition of A bases and linkers, target fragment selection, and PCR. The library was subjected to pair-end 150 bp (PE 150) sequencing using the Illumina HiSeq 4000 platform (Illumina HiSeq 4000 System, RRID:SCR_016386). The data was subjected to quality control and used for analysis. The results showed that a total of 47.42 Gb of data were obtained (Table 1). Ten thousand reads were randomly selected to search the NT database using BLAST, and 90.62% of the reads were mapped to the *Malus* and *Pyrus* genomes. No reads were mapped to microorganisms or animals, which confirmed that the sample was free from contamination. The GC content of the genome is estimated to be approximately 39.65%.

A kmer is an oligonucleotide sequence of length k extracted from the sliding windows of sequencing data. Under the premise of a uniform distribution of sequencing reads, the following formula is obtained:

$$\text{Genomic size} = \frac{\text{total number of bases}}{\text{average sequencing depth}} = \frac{\text{total kmer}}{\text{average kmer depth}}$$

A kmer map of k=21 was constructed using the 350 bp library data (Fig. 2) for the evaluation of genome size, the repeat sequence ratio, and heterozygosity. The main peak corresponding to the kmer depth was 55, which was the average kmer depth. A sequence in which the kmer depth appeared to be more than twice the depth of the main peak (depth value, 111) was considered a repeat sequence. A kmer depth was half of the main peak (depth value, 27.5) indicated that the sequence was heterozygous. The total number of kmers obtained from the sequencing data was 41,072,179,362. After the removal of kmers with an abnormal depth, a total of 39,711,658,265 kmers were used for genome size estimation, and the calculated genome length was approximately 710.83 Mbp, which was consistent with the size of 654.40 Mbp estimated by flow cytometry [6].

According to the kmer distribution, the estimated repeat sequence ratio was approximately 54.56%. There was no obvious heterozygous peak, and the heterozygosity was low, at 0.48%.

Nanopore, Hi-C and RNA sequencing

Genomic DNA was extracted and sequenced following the instructions of the Ligation Sequencing Kit (Nanopore, UK). The DNA was purified, and its quantity was assessed with a Qubit 2.0 Fluorometer (Thermo Fisher, USA). The DNA was randomly sheared, and fragments of ~20 kb were enriched and purified. Damaged DNA and ends were enzymatically repaired with the NEBNext End Repair/dA-Tailing Module (NEB, UK). Then, a 20-kb library was constructed and sequenced on the Nanopore PromethION platform using two flow cells, according to the manufacturer’s protocols (PromethION, RRID:SCR_017987). Approximately 106.23 Gb of data was obtained. After data quality control, the final data volume was 100.10 Gb (Table 1). A Hi-C sample library was constructed from genomic DNA from the fresh leaves of the loquat [7]. The main procedures included cross-linking the DNA, restriction enzyme digestion, end repair, DNA cyclization, and DNA purification. The library was sequenced on the Illumina HiSeq 4000 platform [8]. A total of 67.25 Gb of clean data was obtained, and the Q30 was 94.38%. RNA-seq samples were obtained by mixing equal amounts of RNA extracted from each tissue (leaf, fruit, bud, root, and branch) and used for library construction. After sequencing on the Illumina HiSeq 4000 platform, we obtained 11.06 Gb of sequencing data (Table 1).

Genome assembly based on Nanopore and Hi-C data

In Nanopore sequencing data, the N50 value and the average length of the reads reached 18.06 and 16.15 Kb, respectively (Additional Table S1). According to the estimated genome size (710.83 Mbp), the sequencing depth was 131.69X. First, the Nanopore Reads were corrected by the correction function in Canu (Canu, RRID:SCR_015880, v1.4) [9]. Second, the corrected reads (6,198,187 reads) were assembled by SMARTdenovo (SMARTdenovo, RRID:SCR_017622) [10] to obtain the draft genome with 597 contigs covering 732.25 MB. Third, Racon (Racon, RRID:SCR_017642) [11] was used to calibrate the draft genome with Nanopore reads through three rounds, and the genome size was corrected to 753.38 Mb. Fourth, Pilon (Pilon, RRID: SCR_014731, v1.21) [12] was used to calibrate the draft genome with short genome reads from the Illumina HiSeq 4000 platform through 3 rounds with error radio of 1.64%, 0.07%, and 0.01%, respectively. Finally, the total length of the draft genome sequence was 760.10 Mb, composed of 597 contigs, and the

contig N50 was 5.02 Mb.

BWA (BWA, RRID:SCR_010910, v0.7.15) [13] was used to map the Hi-C short reads obtained from the Illumina HiSeq platform against the draft genome. The comparison mode was ‘aln’, and the other parameters were set to the defaults. The number of unique mapped read pairs was 135,734,826, which accounted for 60.42% of the total read pairs. These unique read pairs were evaluated by HiC-Pro (HiC-Pro, RRID: SCR_017643) [14] to compare the valid interaction pairs and the invalid interaction pairs mapped to the draft genome. The result showed that the percent of valid interaction pairs was 73.97%. In conclusion, the Hi-C library exhibited high quality. The contigs were split at a length of 50 Kb and reassembled according to Hi-C data. A position that could not be restored to the original assembly sequence was listed as a candidate error region, and the low Hi-C coverage depth in this region confirmed this error. After correction, 819 contigs (760.10 MB) were identified. LACHESIS (LACHESIS, RRID:SCR_017644) [15] was used to group, sort, and orient all contigs. A total of 800 contigs (757.53 MB, 99.66%) could be mapped to 17 chromosomes. In the assembly process, the order and direction of 305 contigs were clear, accounting for 676.24 Mb (88.97%), which were assembled to the chromosomes (Additional Table S2). Finally, 17 chromosomes and 514 unplaced scaffolds were obtained in the chromosome-level genome (Table 2). The scaffold N50 was 39.7 Mb.

Evaluation of assembly quality

The integrity of the assembled genome was assessed. First, BWA (BWA, RRID: SCR_010910, v0.7.15) [13] was used to compare the short reads obtained from the Illumina HiSeq sequencing data with the reference genome. The percent of reads mapped to the reference genome was up to 99.69%. Second, CEGMA (CEGMA, RRID:SCR_015055, v2.5) [16] was used to assess the integrity of 458 conserved core genes for eukaryotes, and 451 (98.47%) genes were present in the assembled genome. Third, the Benchmarking Universal Single-Copy Orthologs database (BUSCO, RRID:SCR_015008, v2.0) [17] was used to assess the completeness of gene regions, which contained 1,440 conserved core genes. The results showed that 96.81% of the plant single-copy orthologues were complete. Complete single-copy and multicopy genes accounted for 64.65% and 32.15% of the genes, respectively. These results therefore indicating that the loquat genome assembly presented high quality and coverage.

Genome annotation

LTR_FINDER (LTR_FINDER, RRID:SCR_015247) [18] and RepeatScout (RepeatScout, RRID:SCR_014653) [19] were used for the *de novo* prediction of repetitive sequences in the loquat genome, and all isolated sequences were then classified by PASTEClassifier (PASTEClassifier, RRID:SCR_017645) [20] and mapped to the Repbase database using RepeatMasker (RepeatMasker, RRID:SCR_012954) [21]. A total of 449.72 Mb of repeat sequences were identified, accounting for 59.17% of the genome size (Table 3). Among these repeat sequences, 48.6% (369.44 Mb) and 9.65% (73.34 Mb) were predicted as Class I transposons and Class II retrotransposons (Table 3). In Class I, *copia* and gypsy retrotransposons account for 15.84% (120.38 Mb) and 26.28% (199.73 Mb) of the retrotransposons, respectively. In Class II, TIR and helitron transposons account for 6.85% and 1.96% of the transposons, respectively. The results showed that retrotransposons accounted for a large proportion of the loquat genome.

Protein-coding genes were predicted based on three different strategies, including de novo prediction, homologous species prediction, and Unigene prediction. Genscan (Genscan, RRID:SCR_012902) [22], Augustus (Augustus, RRID:SCR_015981, v2.4) [23], GlimmerHMM (GlimmerHMM, RRID:SCR_002654, v3.0.4) [24], GeneID (GeneID, RRID:SCR_002473, v1.4) [24], and SNAP (SNAP, RRID:SCR_005501) [25] were used for *de novo* prediction (Additional Table S3). GeMoMa (GeMoM, RRID:SCR_017646, v1.3.1) [27] was used for prediction based on homologous species. The transcripts were assembled by using Hisat (Hisat, RRID:SCR_015530, v2.0.4) [28] and Stringtie (StringTie, RRID:SCR_016323, v1.2.3) [29] with default parameters based on RNA-seq data [30], and then TransDecoder (TransDecoder, RRID:SCR_017647) [31], GeneMarkS-T (GeneMarkS-T, RRID:SCR_017648, v5.1) [32] and PASA (PASA, RRID:SCR_014656, v2.0.2) [33] were used for gene prediction (Additional Table S3). Finally, EvidenceModeler (EVM, RRID:SCR_014659, v1.1.1) [34] was used to integrate the prediction results obtained through the above three methods. The Venn diagram showed that 27,685 genes were predicted via all three strategies (Additional Fig. S1), and 45,743 genes corresponding to 160.87 Mb were predicted (Additional Table S3). To better understand gene function, we searched all 45,743 protein-coding genes against protein databases, including InterProScan, KEGG, SwissProt, and TrEMBL. The results showed that 98.69% of the genes could be annotated from these databases. The distribution of repetitive sequences and protein-coding genes is shown in Fig. 3B, 3C.

Based on the Rfam database [35], Blastn (Blastn, RRID:SCR_001598) was used for genome-wide alignment to identify microRNAs and rRNAs. tRNAs were predicted with tRNAscan-SE (tRNAscan-SE, RRID:SCR:010835) [36]. A total of 656 tRNAs, 6,211 rRNAs, and 121 miRNAs were predicted. GeneWise (GeneWise, RRID:SCR_015054) [37] was used to identify immature stop codons and frameshift mutations in the predicted genes to obtain pseudogenes, and 7,642 pseudogenes were obtained.

Gene clusters and duplication

The protein sequences of *E. japonica* and six related species (*Malus domestica*, *Prunus persica*, *Pyrus communis*, *Rubus occidentalis*, *Rosa chinensis*, and *Fragaria vesca*) were compared to analyze the duplication of genes and the classification of species-specific genes between species. The genomes of all related species were downloaded from the Genome Database for Rosaceae. OrthoMCL (OrthoMCL, RRID:SCR_007839) [38] was used to identify the gene families unique to all species. In *E. japonica*, 45,743 genes were grouped into 17,333 gene families (Table 4), which was a greater number than in the other species. The number of genes and gene families in *E. japonica* was similar to that in *P. communis*, which exhibited 45,217 genes and 16,875 gene families. *E. japonica* presented 665 unique families, suggesting that these families were special in the loquat genome. The classification of genes showed that the number of single-copy genes in loquat was lower than in the other species, and 1849 single-copy genes were identified. The loquat and pear presented large numbers of multiple-copy genes (Fig. 4A). CAFE (CAFE, RRID:SCR_005983) was used to study gene family expansion [39]. The results showed that 182 genes were expanded in *E. japonica* compared with *M. domestica* and *P. communis*, including the NB-ARC domain, transposase family tnp2, and the Myb/SANT-like DNA-binding domain (Additional Table S4).

Due to limited computing power, fifty-one single-copy genes in loquat and six related species were randomly selected to construct a phylogenetic tree using MEGA (MEGA, RRID:SCR 000667, v7.0.26). The method of maximum-likelihood–based phylogenetic analyses was performed with *Rubus occidentalis* as the outgroup. The results indicated that the *Eriobotrya* shows a close relationship with the *Malus* and *Pyrus* (Fig. 4). To further investigate the divergence times of these species, the RelTime model was used. Fossil records were downloaded from the TIMETREE website [40] and used to calibrate the results. The divergence time of *Malus* and *Prunus* was set to 45.50 million years ago. The results showed that the loquat diverged from *Malus* ~6.76 million

years ago (Fig. 4B).

4DTv (4-fold degenerate synonymous sites of the third codons) values were calculated according to the homologous gene pairs between two species or within the species itself. The 4DTv distribution map revealed two whole-genome replication events. A divergence peak value (4DTv ~ 0.01) was observed for *E. japonica* -vs- *P. communis* in the map, and low values were found in *E. japonica* -vs- *R. chinensis* (Fig. 4C), which suggested that the divergence of *E. japonica* and *P. communis* occurred relatively later than the divergence of *E. japonica* and *R. chinensis*. In a self-alignment of the chromosomes based on gene synteny, a peak value (0.05) was found among the 4DTv values, suggesting that a whole-genome or large-fragment duplication occurred in the *Eriobotrya* genome. *Eriobotrya* and *Malus* presented clear 2:2 synteny, implying that they shared a common whole-genome duplication event.

Chromosome evolution between the *Malus*, *Prunus*, and *Eriobotrya* genomes

The evolution of the *Eriobotrya* chromosomes and gene collinearity was evaluated using MCScan (MCScan, RRID:SCR_017650, v0.8). The chromosomes of *Prunus* and *Malus* were used as reference genomes. A total of 26,557 and 40,928 gene pairs were found in the inter-genomic comparisons of *Eriobotrya* vs. *Prunus* and *Eriobotrya* vs. *Malus*, respectively. The alignments of syntenic chromosomes were visualized between *Malus*, *Prunus*, and *Eriobotrya* (Fig. 5A). There were fewer scattered points in *Eriobotrya* vs. *Malus* than in *Eriobotrya* vs. *Prunus*, suggesting a close relationship between *Eriobotrya* and *Malus*. The frequency of large-scale fragment rearrangements was found among *Malus*, *Prunus*, and *Eriobotrya*, including inversions and translocations (Fig. 5B). In the comparison of *Prunus* and *Eriobotrya*, the Sac1, 4, and 8 chromosomes of *Prunus* were found to be duplicated (Fig. 5A). Sac1 was divided into LG07/LG08 and LG06/LG15 in *Eriobotrya.* Sac4 and Sac8 were combined and formed LG01 and LG02. Sac5 was not duplicated and formed LG14 in *Eriobotrya*, suggesting that the other copy of Sac5 was lost in the whole-genome duplication. In the comparison of *Malus* and *Eriobotrya*, C05 and C10 in *Malus* were combined and formed LG01 and LG02 in *Eriobotrya*. C09 and C17 formed LG11 and LG13. This result suggested that fragment rearrangements occurred widely on the chromosomes of *Malus* and *Eriobotrya*. These findings implied that *Malus*, *Prunus*, and *Eriobotrya* shared some chromosome regions and that extensive chromosome rearrangements occurred. Overall, these findings provide new insight into the evolution of *Eriobotrya* chromosomes.

Conclusion

To our knowledge, this is the first report of the chromosome-level genome assembly of *E. japonica* using the third-generation sequencing technology of Nanopore and High-through chromosome conformation capture. A total of 45,743 high-quality protein-coding genes were annotated by integrating the results from 3 different methods, including de novo prediction, homologous species prediction, and Unigene prediction. Phylogenetic analysis indicated that *Eriobotrya* is closely related to *Malus*. The analysis showed that a whole-genome or large-fragment duplication occurred in the *Eriobotrya* genome. The chromosomal rearrangement was found in *Eriobotrya* and *Malus*. This work provides valuable chromosome-level genomic data for loquat and important genomic data for studying loquat traits.

Abbreviations

4DTv: 4-fold degenerate synonymous sites of the third codons; BLAST: Basic Local Alignment Search Tool; bp: base pairs; Gb: gigabase pairs; GO: Gene Ontology; Hi-C: high-throughput chromosome conformation capture; HiSeq: high-throughput sequencing; kb: kilobase pairs; KEGG: Kyoto Encyclopedia of Genes and Genomes; Mb: megabase pairs; miRNA: MicroRNA; RNA-seq: RNA-sequencing; rRNA: ribosomal RNA; TrEMBL: a database of translated proteins from European Bioinformatics Institute; tRNA: Transfer RNA.

Conflict of interest

The authors declare that they have no competing interests.

Author contributions

Shuang Jiang performed the experiments and wrote the manuscript. Haishan An helped to collect the samples and revise the manuscript. Fangjie Xu helped to analyze the data and revise the manuscript. Xueying Zhang involved in designing the research and revised the manuscript. All authors read and approved the manuscript.

Availability of supporting data and materials

The raw sequence data have been deposited in NCBI under project accession No. PRJNA579885. The run of clean reads in RNA-seq, HiC, Illumina HiSeq, and Nanopore were deposited in Genome Sequence Archive in NCBI under the Bioproject accession number PRJNA579885 (SRR10377313~ SRR10377316). Data was also submitted the BIG Data Center, Beijing Institute of Genomics (BIG), Chinese Academy of Sciences under BioProject number PRJCA001836. For genome assembly data, the accession number is GWHAAZU00000000 in the BGI Genome Warehouse. The run of clean reads of Nanopore, Illumina HiSeq, HiC, and RNA-seq data were deposited in Genome Sequence Archive in BIG under the accession number of CRR078404~CRR078407. All supporting data and materials are available in the *GigaScience* GigaDB database [41].

Funding

This work was financed by a Grant for Agriculture Applied Technology Development Program from Shanghai Agriculture Committee (2015-6-2-2) and a Grant from the National Natural Science Foundation of China (No. 31701886).

References:

1. Lindley J. *Eriobotrya japonica*. Transactions of the Linnean Society of London 1821;13(1):102.
2. Yang X, Glakpe K, Lin S, et al. Taxa of plants of genus Eriobotrya around the world and native to Southeastern Asia. Joumal of Fruit Science 2005;**22**(1):55-9 (in Chinese).
3. Li GF, Zhang ZK, Lin SQ. Origin and evolution of eriobotrya. Acta Hortic 2011;**887**:33-7.
4. Lo EY, Donoghue MJ. Expanded phylogenetic and dating analyses of the apples and their relatives (Pyreae, Rosaceae). Mol Phylogenet Evol 2012;**63**(2):230-43.
5. Doyle JJ, Doyle JL. A rapid DNA isolation procedure for small quantities of fresh leaf tissue. Phytochem Bull 1987;**19**:11-5.
6. Zhang ZK, Wang YQ, Lin SQ, Du K. Ploidy identification of loquats for genome sequencing project by flow cytometry. Journal of Fruit Science 2012;**29**(3):498-504 (in chinese).
7. Dekker J, Marti-Renom MA, Mirny LA. Exploring the three-dimensional organization of genomes: interpreting chromatin interaction data. Nat Rev Genet 2013;**14**(6):390-403.
8. Scott Edmunds (2018). Hiseq 4000 Sequencing protocol. **protocols.io** dx.doi.org/10.17504/protocols.io.q58dy9w
9. Koren S, Walenz BP, Berlin K, et al. Canu: scalable and accurate long-read assembly via adaptive k-mer weighting and repeat separation. Genome Res 2017;**27**(5):722-36.
10. Ruan J. https://github.com/ruanjue/smartdenovo. Accessed 20 August 2019.
11. Vaser R, Sovic I, Nagarajan N, Sikic M. Fast and accurate de novo genome assembly from long uncorrected reads. Genome Res 2017;**27**(5):737-46.
12. Walker BJ, Abeel T, Shea T, et al. Pilon: an integrated tool for comprehensive microbial variant

detection and genome assembly improvement. Plos One 2014;**9**(11):e112963.

13. Li H, Durbin R. Fast and accurate short read alignment with Burrows-Wheeler transform. Bioinformatics 2009;**25**(14):1754-60.
14. Servant N, Varoquaux N, Lajoie BR, et al. HiC-Pro: an optimized and flexible pipeline for Hi-C data processing. Genome Biol 2015;**16**:259.
15. Burton JN, Adey A, Patwardhan RP, et al. Chromosome-scale scaffolding of de novo genome assemblies based on chromatin interactions. Nat Biotechnol 2013;**31**(12):1119-25.
16. Parra G, Bradnam K, Korf I. CEGMA: a pipeline to accurately annotate core genes in eukaryotic genomes. Bioinformatics 2007;**23**(9):1061-7.
17. Simao FA, Waterhouse RM, Ioannidis P, et al. BUSCO: assessing genome assembly and annotation completeness with single-copy orthologs. Bioinformatics 2015;**31**(19):3210-2.
18. Xu Z, Wang H. LTR_FINDER: an efficient tool for the prediction of full-length LTR retrotransposons. Nucleic Acids Res 2007;**35**(Web Server issue):W265-8.
19. Price AL, Jones NC, Pevzner PA. De novo identification of repeat families in large genomes. Bioinformatics 2005;**21**(Suppl 1):i351-8.
20. Hoede C, Arnoux S, Moisset M, et al. PASTEC: an automatic transposable element classification tool. Plos One 2014;**9**(5):e91929.
21. Tarailo- Graovac M, Chen N. Using RepeatMasker to identify repetitive elements in genomic sequences. Curr Protoc Bioinformatics 2009;**4**(10):11-4.
22. Burge C, Karlin S. Prediction of complete gene structures in human genomic DNA. J Mol Biol 1997;**268**(1):78-94.
23. Stanke M, Waack S. Gene prediction with a hidden Markov model and a new intron submodel. Bioinformatics 2003;**19**(Suppl 2):i215-25.
24. Majoros WH, Pertea M, Salzberg SL. TigrScan and GlimmerHMM: two open source ab initio eukaryotic gene-finders. Bioinformatics 2004;20(16):2878-9.
25. Blanco E, Parra G, Guigó R. Using geneid to identify genes. Curr Protoc Bioinformatics 2007;**4**(3):1-28.
26. Korf I. Gene finding in novel genomes. BMC Bioinformatics 2004;**5**:59.
27. Keilwagen J, Hartung F, Paulini M, et al. Combining RNA-seq data and homology-based gene prediction for plants, animals and fungi. BMC Bioinformatics 2018;**19**(1):189.
28. Kim D, Langmead B, Salzberg SL. HISAT: a fast spliced aligner with low memory requirements. Nat Methods 2015;**12**(4):357-60.
29. Pertea M, Pertea GM, Antonescu CM, et al. StringTie enables improved reconstruction of a transcriptome from RNA-seq reads. Nat Biotechnol 2015;**33**(3):290-5.
30. Pertea M, Kim D, Pertea GM, et al. Transcript-level expression analysis of RNA-seq experiments with HISAT, StringTie and Ballgown. Nat Protoc. 2016;**11**(9):1650-67.
31. Haas B, Papanicolaou A. TransDecoder (Find Coding Regions Within Transcripts) http://transdecoder.github.io. Accessed 20 August 2019.
32. Tang S, Lomsadze A, Borodovsky M. Identification of protein coding regions in RNA transcripts. Nucleic Acids Res 2015;**43**(12):e78.
33. Campbell MA, Haas BJ, Hamilton JP, et al. Comprehensive analysis of alternative splicing in rice and comparative analyses with Arabidopsis. BMC Genomics 2006;**7**:327.
34. Haas B, Salzberg S, Zhu W, et al. Automated eukaryotic gene structure annotation using EVidenceModeler and the program to assemble spliced alignments. Genome Biol 2008;**9**:R7.

35. Griffiths-Jones S, Moxon S, Marshall M, et al. Rfam: annotating non-coding RNAs in complete genomes. Nucleic Acids Res 2005;**33**(Database issue):D121-4.
36. Lowe TM, Eddy SR. tRNAscan-SE: a program for improved detection of transfer RNA genes in genomic sequence. Nucleic Acids Res 1997;**25**(5):955-64.
37. Birney E, Clamp M, Durbin R. GeneWise and Genomewise. Genome Res 2004;14(5):988-95.
38. Li L, Stoeckert CJ, Roos DS. OrthoMCL: identification of ortholog groups for eukaryotic genomes. Genome Res. 2003;**13**(9):2178-89.
39. De Bie T, Cristianini N, Demuth JP, Hahn MW. CAFE: a computational tool for the study of gene family evolution. Bioinformatics. 2006;**22**(10):1269-71.
40. TimeTree. www.timetree.org. Accessed 20 August 2019.
41. Jiang S; An H; Xu F; Zhang X (2020): Supporting data for "The chromosome-level genome assembly and annotation of the loquat (*Eriobotrya japonica*) genome" *GigaScience* Database. http://dx.doi.org/10.5524/100711.

Figure legends

Figure 1 Picture of a loquat variety, seventh star (*Eriobotrya japonica*).

Figure 2 The Kmer analysis (K=23) of *Eriobotrya japonica* genome characteristics.

Figure 3 Summary of the *de novo* genome assembly and sequencing analysis of *Eriobotrya japonica*. A, Chromosome number; B, numbers of repeat sequences per Mb; C, numbers of protein coding genes per Mb; and D paralogous relationships between E. *japonica* chromosomes.

Figure 4 The genome evolution of *Eriobotrya*. (A) Comparison of copy numbers in gene clusters of *Eriobotrya* genomes and six related species genomes. Onecopy, single copy genes. Multicopy, multicopy genes. Special_gene, species-specific genes. Other_gene, the rest of clustered genes other than the above genes. Unclusternum, unclustered genes. (B) Constructed phylogenetic tree and divergence time estimation. (C) 4DTv analyses in *Eriobotrya* and related species.

Figure 5 The chromosomes collinearity among *Malus*, *Prunus* and *Eriobotrya*. (A) The inter-genomic comparison. (B) The chromosomes map in three species.

Additional files

Additional Figure S1 COG function classification of all unigenes.

Additional Table S1 The sequence length of reads in Nanopore.

Additional Table S2 The details of the distribution of each chromosome sequences.

Additional Table S3 Gene prediction result statistics.

Additional Table S4 The number of expansion gene in *E. japonica* compared with *M. domestica* and *P. communis*.

Table 1: Sequencing data used for loquat genome assembly and annotation

Sequencing type	Platform	Library size (bp)	Clean data (Gb)	Application
Genome short reads	Illumina HiSeq 4000	350	47.42	Genome survey and assessment
Nanopore reads	Nanopore platform	20000	100.10	Contig assembly
Hi-C reads	Illumina HiSeq 4000	300-700	67.25	Chromosome construction
Transcriptome short reads	Illumina HiSeq 4000	200-500	11.06	Genome annotation and assessment

Table 2 Assembly statistics

	Software	Assembly level	Number of sequences	N50 (Mb)	size (Gb)
Nanopore	Smartdenovo, Racon, and Pilon	contig	597	5.0	760.1
Nanopore and Hi-C	Lachesis	chromosome	17 + 514[a]	39.7	676.2 + 83.9

[a]There are 514 unplaced scaffolds in the final chromosome-level assembly. These unplaced contigs comprise ~10.73% of total bases in the genome assembly size.

Table 3 Repeat sequences in the loquat genome.

Type	Number	Length	Rate(%)
ClassI	457393	369440909	48.6
ClassI/DIRS	11457	9761251	1.28
ClassI/LINE	26529	8851756	1.16
ClassI/LTR	36969	15617403	2.05
ClassI/LTR/Copia	141908	120380193	15.84
ClassI/LTR/Gypsy	183863	199727884	26.28
ClassI/PLE|LARD	54589	14439960	1.9
ClassI/SINE	812	155412	0.02
ClassI/SINE|TRIM	7	3188	0
ClassI/TRIM	1223	497670	0.07
ClassI/Unknown	36	6192	0
ClassII	210159	73341918	9.65
ClassII/Crypton	7	403	0
ClassII/Helitron	45852	14912320	1.96
ClassII/MITE	561	159816	0.02
ClassII/Maverick	405	107504	0.01
ClassII/TIR	140384	52101491	6.85
ClassII/Unknown	22950	6060384	0.8
PotentialHostGene	2021	451961	0.06
SSR	346	66302	0.01
Unknown	26488	6427210	0.85
Total	669919	449728153	59.17

Table 4 The statistics of gene family classification in seven species.

Species	Total genes	Cluster number	Total family	Unique family
E. japonica	45,743	39,294	17,333	665
M. domestica	28,306	20,426	12,797	365
P. communis	45,217	32,764	16,875	819
P. persica	26,873	22,583	14,969	310
R. occidentalis	33,253	24,641	15,479	1,241
F. vesca	24,034	21,789	14,859	196
R. chinensis	30,214	26,705	15,326	473

Figure 1

Click here to access/download;Figure;Fig 1.tif

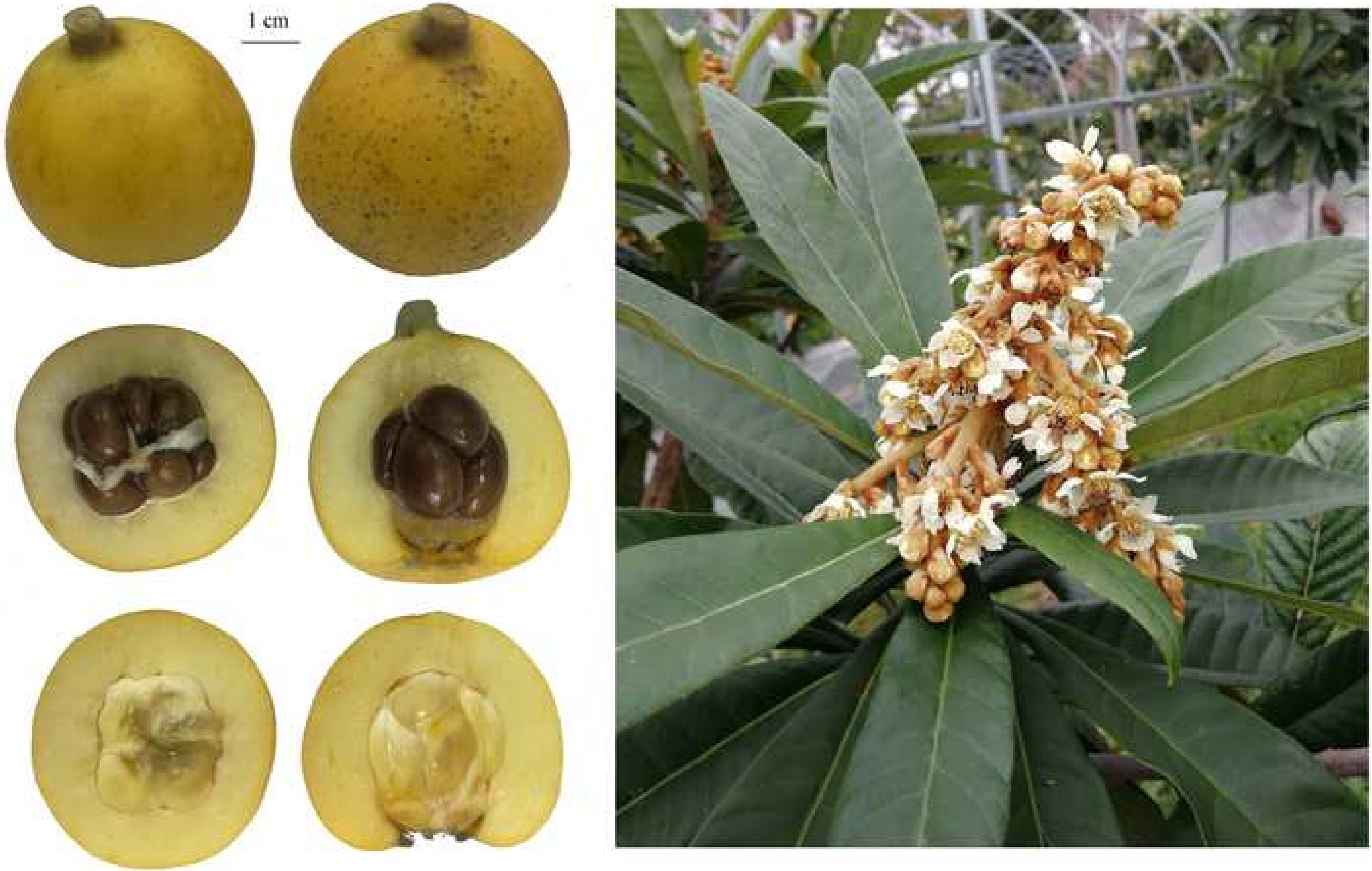

Figure 2

Click here to access/download;Figure;Fig 2.png

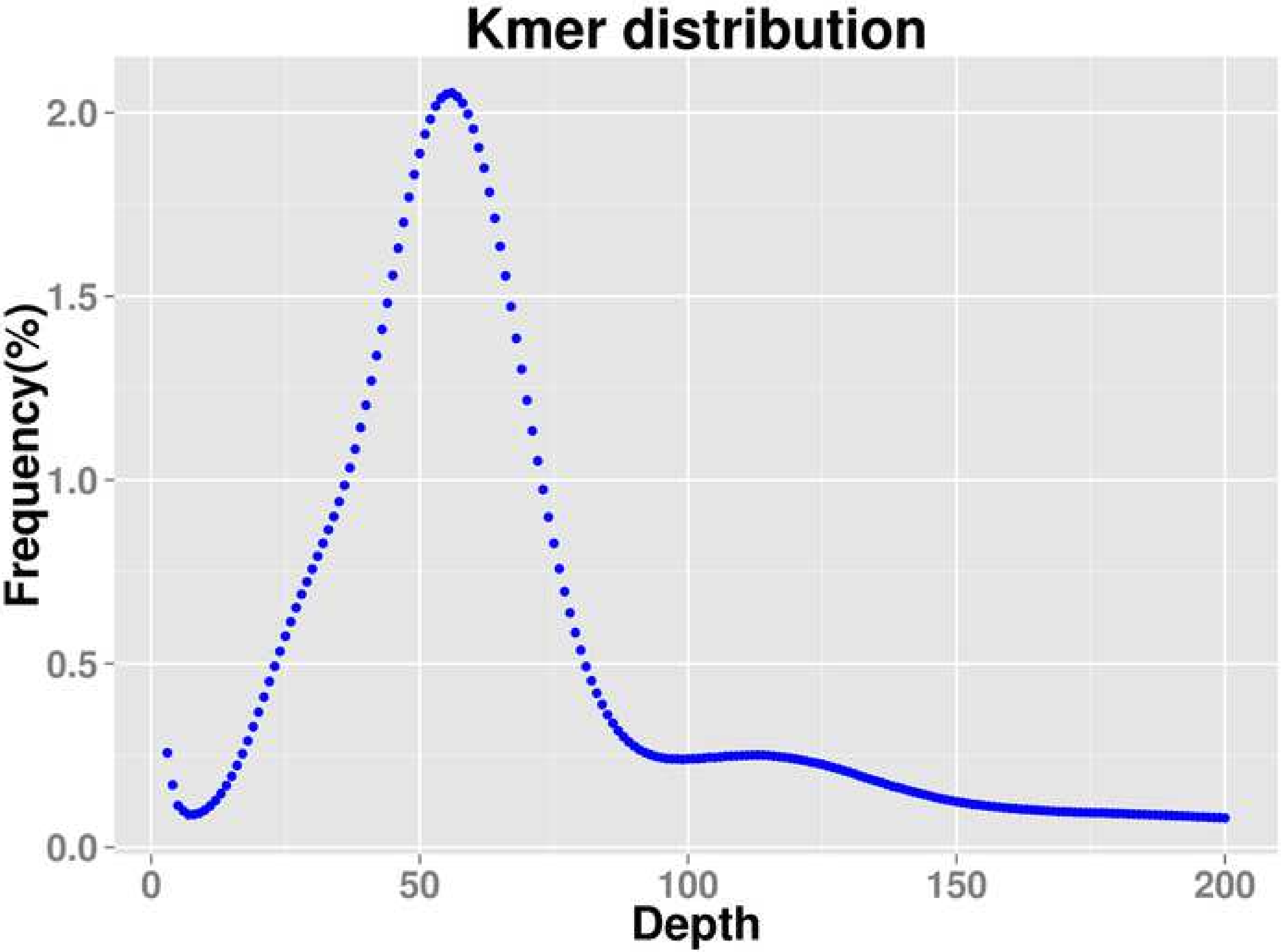

Figure 3

Click here to access/download;Figure;Fig 3.png

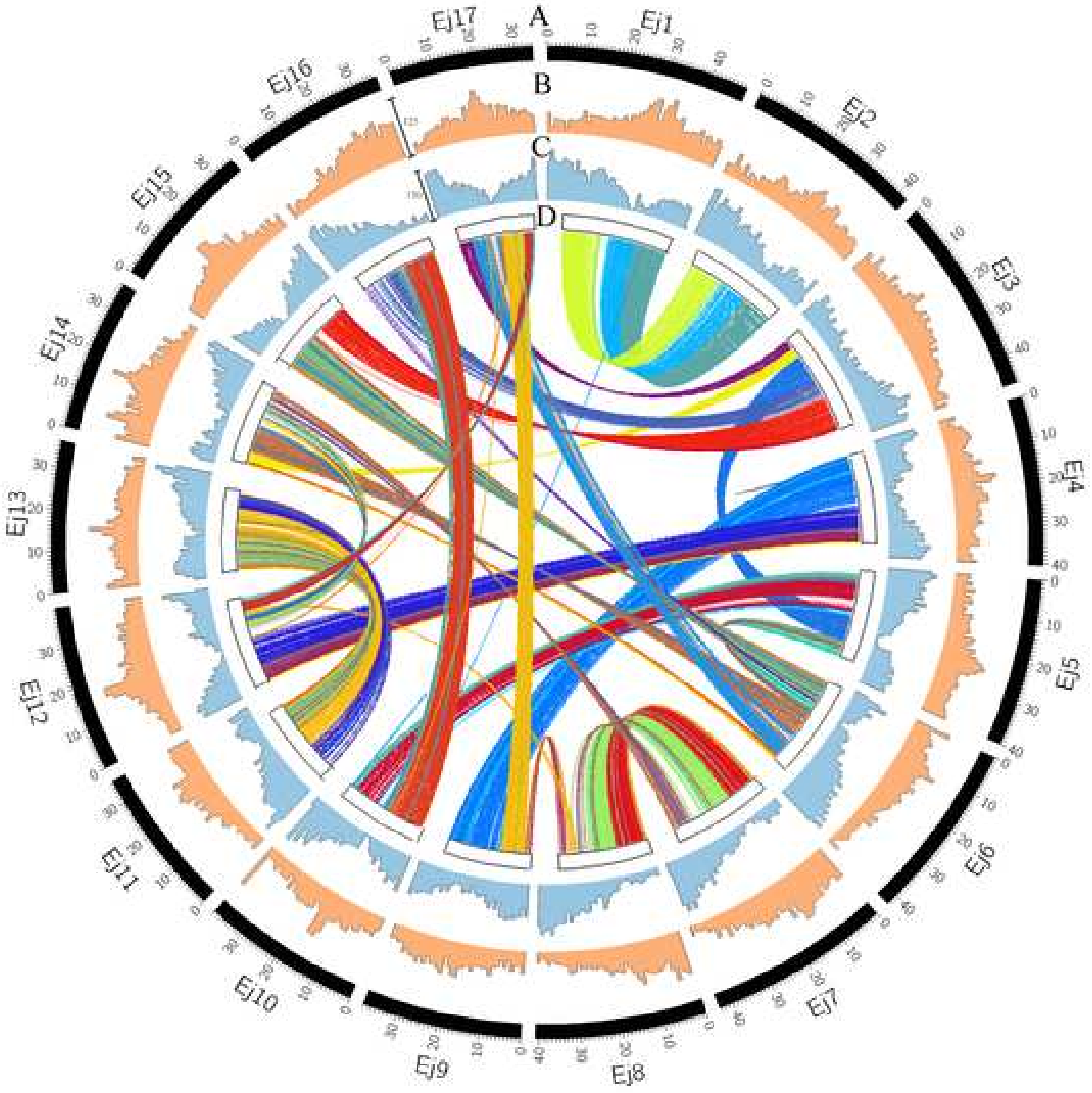

Figure 4

Click here to access/download;Figure;Fig 4.tif

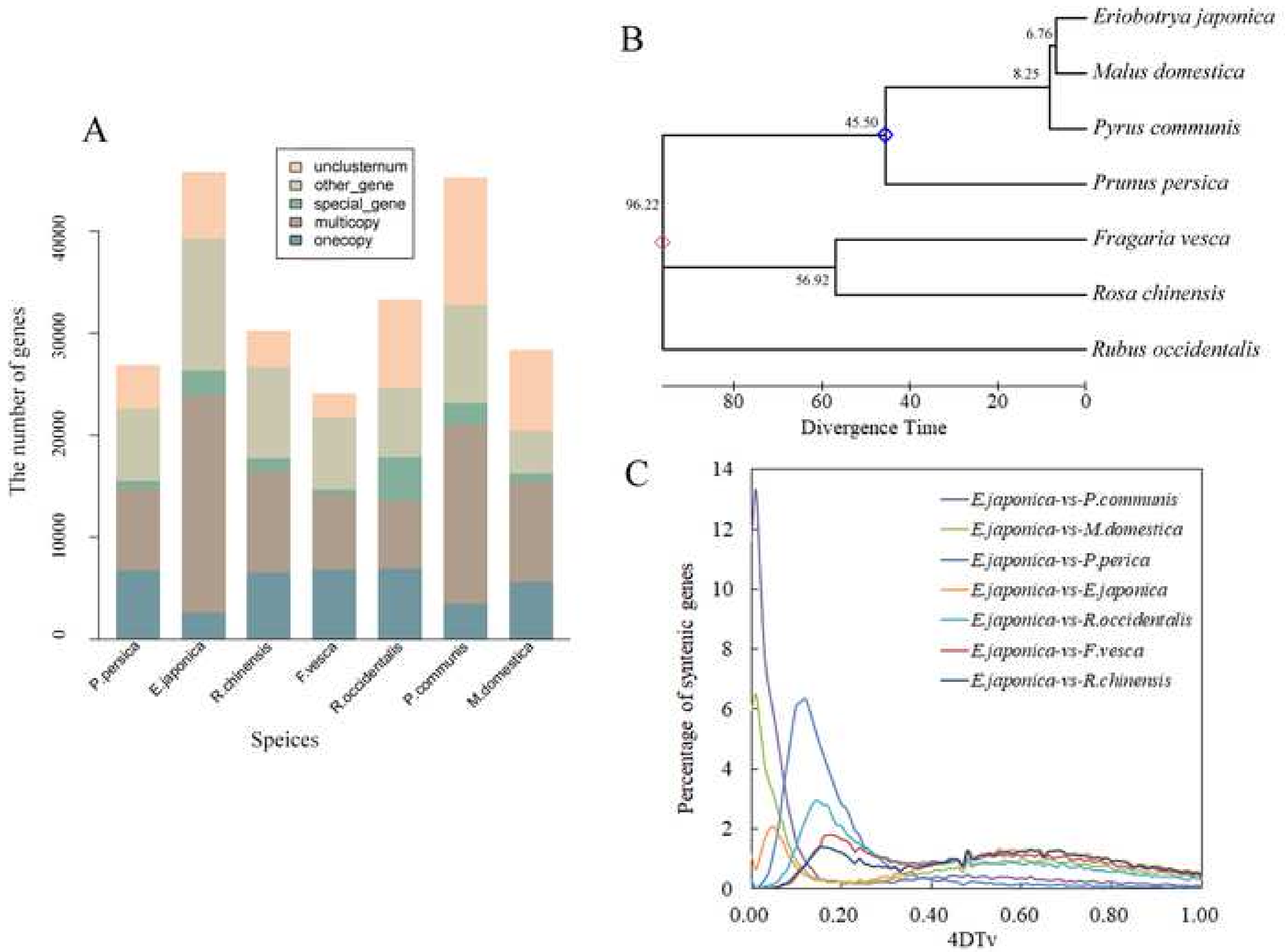

Figure 5

Click here to access/download;Figure;Fig 5.tiff

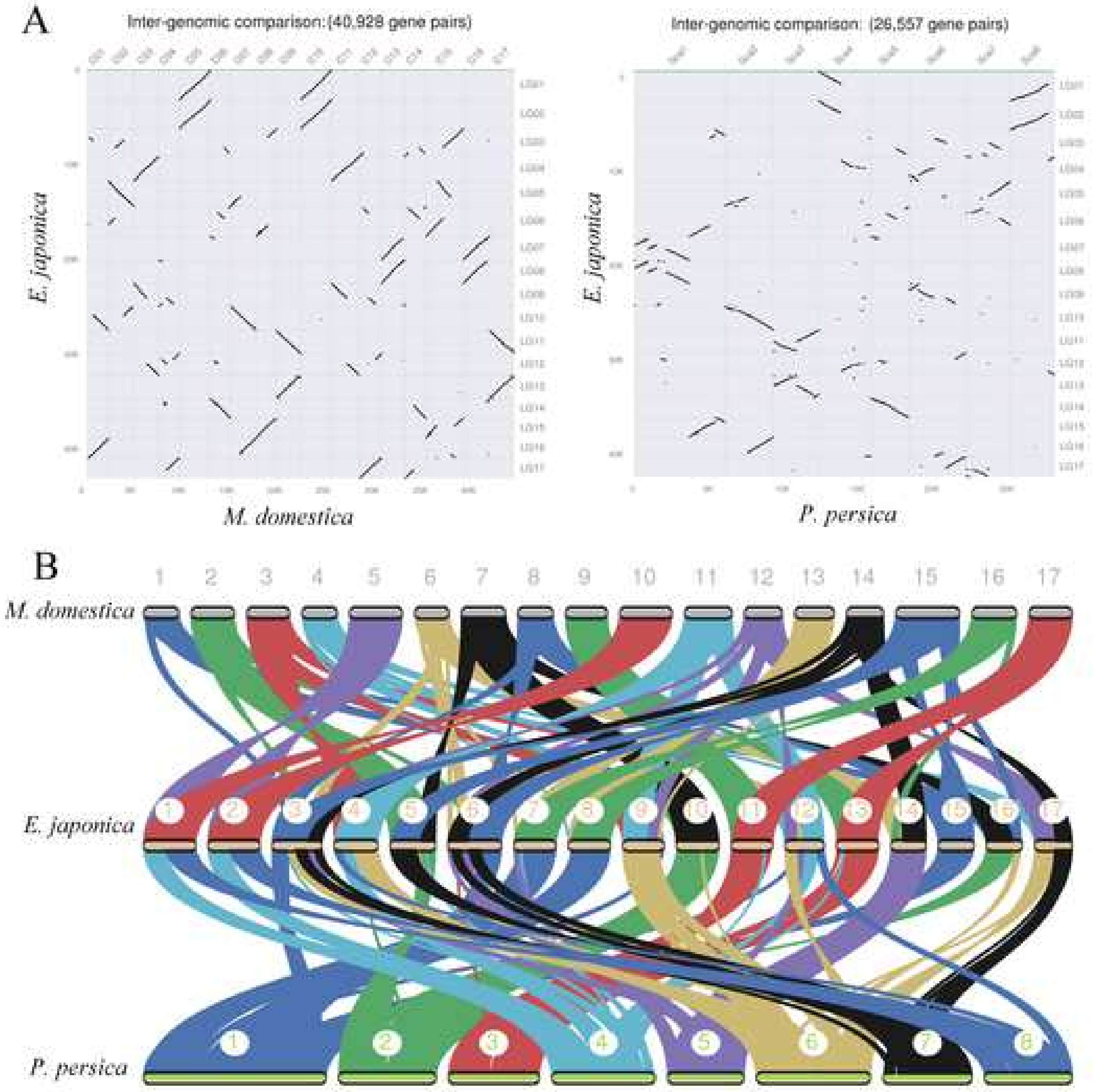

Figure S1

Click here to access/download
Supplementary Material
Fig S1.png

Table S1

Click here to access/download
Supplementary Material
Table S1.xlsx

Table S2

Click here to access/download
Supplementary Material
Table S2.xlsx

Table S3

Click here to access/download
Supplementary Material
Table S3.xlsx

Table S4

Click here to access/download
Supplementary Material
Table S4.xlsx
