# Supplementary figures and images for: Chromosome-level genome assembly and annotation of the loquat (Eriobotrya japonica) genome
Source: Gigascience. 2020 Mar 6;9(3):giaa015. doi: 10.1093/gigascience/giaa015 (PMC7059265; doi:10.1093/gigascience/giaa015)

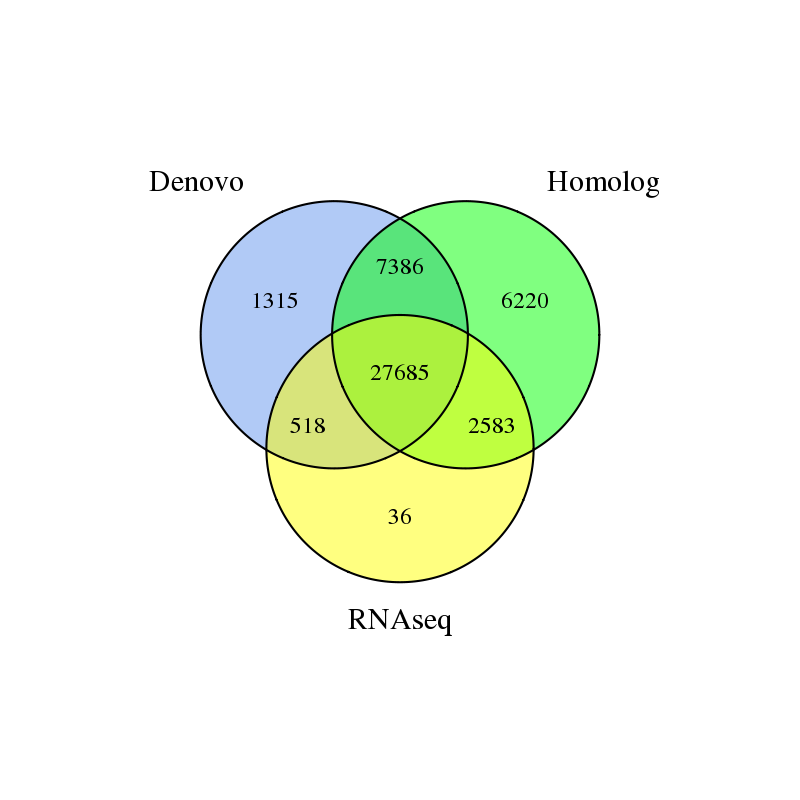

Supplement: giaa015_Supplemental_Figure_and_Tables [file giaa015_supplemental_figure_and_tables.zip › Fig S1.png]
