# Supplementary material for: Chromosome-level genome assembly and annotation of the loquat (Eriobotrya japonica) genome
Source: Gigascience. 2020 Mar 6;9(3):giaa015. doi: 10.1093/gigascience/giaa015 (PMC7059265; doi:10.1093/gigascience/giaa015)
Supplement: giaa015_Response_to_Reviewer_Comments_Original_Submission [file giaa015_response_to_reviewer_comments_original_submission.pdf]

Author's Response To Reviewer Comments

Close

Dear editor:
Thank you very much for your letter. We greatly appreciate the suggestions and comments made by you and the reviewers. Based upon the suggestions and comments, we have revised the manuscript and our manuscript has been edited by AJE English Editing. Specific revisions made on the manuscript are shown below as well as in the new manuscript in red. We hope that the manuscript is now suitable for publication in GigaScience.

Sincerely yours,
Xueying Zhang

The corrections and responses to editor and reviewers' comments and suggestions are as follows:

Editor comments:
Please register any new software application in the bio.tools and SciCrunch.org databases to receive RRID (Research Resource Identification Initiative ID) and biotoolsID identifiers, and include these in your manuscript.

Done. All new software applications have a RRID, and we registered them in bio.tools.

Reviewer reports:
Reviewer #1: Jiang et. al report a chromosome scale genome assembly of the important horticultural crop loquat. They utilized a single-molecule, Nanopore sequencing based approach coupled with Hi-C to generate a high-quality assembly. Loquat and apple have a clear2:2 synteny with a high degree of collinearity, suggesting they have a shared whole genome duplication event and that the loquat genome is high-quality. The resources presented here will be useful for the loquat and Rosaceae research communities as well as the comparative genomics communities. I have a few comments/suggestions that I feel with strengthen the manuscript.

Thank you for your comments. We really appreciate your suggestion.

Major:
1. Significantly more details are needed for the genome assembly section. Based on the methods, it seems like Canu was used to error correct the reads and smartdenovo assembly was used to assemble them into contigs. Then, Racon and Pilon were used to polish the assembly. This is an unusual pipeline to use and it is unclear which data was used in each step. Was the full Canu pipeline used to assemble a draft genome prior to smartdenovo assembly? Or, were error corrected or corrected and trimmed reads used as input for smartdenovo? Pilon requires Illumina short reads for polishing, was the HiSeq4000 data used for this? How many rounds of Racon and Pilon were run on the data? Statistics for each step of the assembly would also be helpful (e.g. how many errors were corrected, the input metrics for smartdenovo, etc.).

Thank you for your comments. A full Canu run includes three stages: correction, trimming, and assembly. We tested the full Canu pipeline to assemble a draft genome. The result showed that the genome size was 280,096,430bp with N50 85,570bp, which was not a good result. We changed our strategy. Canu was only used to correct the Reads by the stage of correction, and then the corrected Reads (genome.correctedReads.fasta) were assembled by SMARTdenovo to obtain the draft genome. The assembly method of Canu+SMARTdenovo was also reported in other studies (e.g. Schmidt MH , Vogel A , Denton AK , et al. 2017. De novo assembly of a new Solanum pennellii accession using Nanopore Sequencing. The Plant Cell, tpc.00521.). Racon used the Nanopore Reads and Pilon used the genome short reads from Illumina HiSeq 4000. The errors were not recorded in Canu and Racon. The error radio of 1.64%, 0.07%, and 0.01% were recorded in 3 rounds in Pilon. We revised this paragraph as "First, the Nanopore Reads were corrected by the correction function in Canu (Canu,

RRID:SCR_015880, v1.4) [8]. Second, the corrected reads (6,198,187 reads) were assembled by SMARTdenovo (SMARTdenovo, RRID:SCR_017622) [9] to obtain the draft genome with 597 contigs covering 732.25 MB. Third, Racon (Racon, RRID:SCR_017642) [10] was used to calibrate the draft genome with Nanopore reads through three rounds, and the genome size was corrected to 753.38 Mb. Fourth, Pilon (Pilon, RRID: SCR_014731, v1.21) [11] was used to calibrate the draft genome with short genome reads from the Illumina HiSeq 4000 platform through 3 rounds with error radio of 1.64%, 0.07%, and 0.01%, respectively. Finally, the total length of the draft genome sequence was 760.10 Mb, composed of 597 contigs, and the contig N50 was 5.02 Mb."

2. The manuscript contains would benefit from heavy editing for clarity.

We really appreciate your suggestion.

Minor:

1. Line 65: "The reads were searched by the NT database, which confirmed that the sample is free from contamination." It is unclear what this means. My interpretation is that a subset of reads were queried against a database using BLAST or another alignment program to identify contaminant sequences. More details should be provided here.

We added a new sentence as "Ten thousand reads were randomly selected to search the NT database using BLAST, and 90.62% of the reads were mapped to the Malus and Pyrus genomes. No reads were mapped to microorganisms or animals, which confirmed that the sample was free from contamination."

2. Line 82. A heterozygosity rate of 0.48 may be low relative compared to other highly heterozygotic species, but it would likely still present a challenge for genome assembly. Smartdenovo assembly will smash haplotypes together but programs like Canu should keep them separate during assembly. Was the full Canu pipeline run on the assembly? If so, how does this compare to the Smartdenovo assembly?

Canu was only used to correct the Reads by the stage of correction. The corrected Reads were assembled by SMARTdenovo to obtain the draft genome.

3. Line 87 Interrupted to sheared

Done.

4. Line 92. A protocol should be referenced for the HiC library construction

We added a reference [7].

5. Line 106. Parameters should be reported for aligning the HiC reads to the genome using BWA.

The comparison mode was 'aln', and the other parameters were default. We added a sentence as "The comparison mode was 'aln', and the other parameters were set to the defaults." in the revised manuscript.

6. Line 111. Interrupted to split

Done.

7. Line 116. It is unclear how 800 contigs were mapped to 17 chromosomes, but only 305 were oriented into the 17 pseudomolecules.

819 contigs (305+495+19, 760.1 Mb) were identified. 305 contigs (676.24 Mb, 88.97%) were capable of determining the order and direction. 495 contigs (81.29 Mb, 10.69%) could be mapped to some chromosomes, but their order and direction were not clear. 19 contigs (2.57 Mb) were not mapped to some chromosomes. 305 contigs account for 88.97% in the whole genome. We added some details in the revised manuscript.

8. Line 148. More details should be provided on how transcripts were assembled and what cutoffs were used. Hisat and Stringtie are listed, but no details are provided.

In most software, we used the default parameters. The usage of Hisat and Stringtie was based on an added reference [29]. The prediction result in this paragraph was shown in Additional Table S3.

9. Line 196. Loquat and apple have clear 2:2 synteny and shared Ks peaks, but it is not explicitly mentioned that they share a common whole genome duplication event.

We added it in the manuscript as "Eriobotrya and Malus presented clear 2:2 synteny, implying that they shared a common whole-genome duplication event".

Reviewer #2: Jiang et al. reported the high quaility genome assembly and annotation for an important fruit tree, Eriobotrya japonica. In my opinion, this study is original, and data analysis have been well planned and conducted. The genomic resources and analysis are valuable for the loquat community and more broader regime of genomics and plant biology. However, there are large spaces for improvement in the English expression. I think editing by a native speaker is necessary.
It could be accepted after minor revision.

Thank you for your comments. We really appreciate your suggestion. Based upon the suggestions and comments, we have revised the manuscript and our manuscript has been edited by AJE English Editing.

Major concern:
Please provide parameters and settings for specific analysis you conducted, especially for the genome assembly part.

We revised this paragraph as "First, the Nanopore Reads were corrected by the correction function in Canu (Canu, RRID:SCR_015880, v1.4) [8]. Second, the corrected reads (6,198,187 reads) were assembled by SMARTdenovo (SMARTdenovo, RRID:SCR_017622) [9] to obtain the draft genome with 597 contigs covering 732.25 MB. Third, Racon (Racon, RRID:SCR_017642) [10] was used to calibrate the draft genome with Nanopore reads through three rounds, and the genome size was corrected to 753.38 Mb. Fourth, Pilon (Pilon, RRID: SCR_014731, v1.21) [11] was used to calibrate the draft genome with short genome reads from the Illumina HiSeq 4000 platform through 3 rounds with error radio of 1.64%, 0.07%, and 0.01%, respectively. Finally, the total length of the draft genome sequence was 760.10 Mb, composed of 597 contigs, and the contig N50 was 5.02 Mb."

Minor comments:
(I am not a native speaker. Here, I pick up specific comments related to generally the language expression)
1. line 11, "It flowered", is it a good expression?

We revised "flowered" as "blossoms".

2. line 19-21, please check this sentence, "The Hi-C ,,,, 39.7 Mb". Do you think Hi-C technology could really do assembly?

We revised this sentence as "The contigs were mapped to chromosomes by using Hi-C technology based on the contacts between contigs"

3. line 22, "analyzed" -> "investigated"

Done.

4. line 23, "the other six Rosaceae" -> "six other Rosaceae"

Done.

5. line 33, "are classified by" -> "were identified by"

Done.

6. line 34, "were" -> "are"

Done.

7. line 40, "The top buds become flowers", why do you want to say this?

We deleted this sentence.

8. line 41, why do you want to use "flowered"?

This word was revised as “blossoms”

9. line 66-67, "Evaluation of the chloroplast of the species ,,, content", why do you want to do that? How did you do that? Is it relevant?

It suggested that the nuclear DNA were sequenced, not chloroplast DNA. It is relevant, and we deleted this sentence.

10. line 82, hard to imagine a simple genome, please define it if you want to describe your assembly as a simple one.

Thank you for your comments. We deleted this sentence.

11. lines 91-92, "A Hi-C ,,, of the loquat", problematic expression. Do you really think the fresh leaf can do Hi-C? Please carefully check the full paper, for this similar problem.

We revised this sentence as “A Hi-C sample library was constructed from genomic DNA from the fresh leaves of the loquat”

12. line 94, "by" -> "with".

This sentence was revised as “The library was sequenced on the Illumina HiSeq 4000 platform.”

13. any reference for "smartdenovo"?

We added a reference [9].

14. line 116, "assembled" -> "assembly"

Done.

15. no need to use "software" so often.

We deleted "software".

16. line 173, please define "unifamiles". and any type there.

We revised "unifamiles" as “unique families”.

17. line 176, if you reported results of gene expansion. Please describe how you did do that?

We added a new sentence here as “CAFE (CAFE, RRID:SCR_005983) was used to study gene family expansion [38]”.

18. line 209-210, "One-to-one corresponding chromosome", hard to explain this. Please define it or make it clear.

It means corresponding one by one. We deleted this sentence.

19. line 216, "Discussion" -> "Conclusion"

Done.

20. line 258, please carefully check your reference list. A lot bugs.

Done

21. line 345, a new title would be "Picture of a loquat variety, seventh star"

Done.

22. Table 1 and 2, could be moved to the supplementary.

Table 1 and 2 showed some data. Can we keep it in the manuscript?

23. Replace Table 3 with Table S3.

Done.

24. line 384, please define "Uni family"

We revised "unifamiles" as "unique families".

25. Figure 4, make it clear, by define the items you used or any other means.

Done. The figures in the pdf file were not original one, pictures lost clarity during conversion. The high quality original figures could be downloaded when you click the website in upper right corner in the picture page.

Reviewer #3: This paper is worth publishing for the Data Note for GigaScience because the authors have constructed a highly accurate genome and gene sequence of loquat. The method is reasonable and the presentation is pretty good. Speaking of greed, since it is clear that the relationship with Malus is relatively close among the fruit trees of the Rosaceae family, so there should be presented some discussion about the traces on the genome that triggered the differentiation of morphological features between both species, I recommend that as it would be done for the future work. The following minor concerns should be corrected before acceptance.

Thank you for your comments. We really appreciate your suggestion.

Minor concerns
1. L58 How to extract the RNA from the collected samples should be described.

We revised this sentences as "The leaves, fruit, buds, roots, and branches were collected for RNA extraction via the CTAB-LiCl method."

2. L63 double-end would be pair-end.

Done.

3. L71 the appearance of the equation is not clear. Please correct the format of the equation.

We enlarged the font size.

4. L86 "quality" have to be "quantity", because the Qubit 2.0 Fluorometer is the device to evaluate the quantity of the DNA/RNA with fluorescence.

Done.

5. L89 How many flow cells have been used with the PromethION platform to acquire about the 106.23 Gb.

Two flow cells have been used. We revised this sentence as "Then, a 20-kb library was constructed and sequenced on the Nanopore PromethION platform in two flow cells".

6. L205 "Sac1, 4, and 8" has been suddenly appeared at the text. Please describe about relationships between the former and later text to be clearly understood, although chromosome scale duplication is

very interesting.

We revise this sentence as “the Sac1, 4, and 8 chromosomes of Prunus were found to be duplicated”

7. L349 "de novo" should be written in italic.

Done.

8. L364 Add period.

Done.

9. L366 Add period.

Done

10. L367 E. japonica should be written in italic.

Done.

11. L375 "Hic" should be "Hi-C"

Done.

12. Figures Provide higher resolutional figures than current version. Because those are not clear. Figure 4A Correct the overlap of the legends on a bar. Figure 5A It is difficult to see the scale of the figure. Please provide higher resolutional figures.

We revised the Figure 4A. The figure in pdf file was not the original one. The high quality original figure could be downloaded when you click the website in upper right corner in the picture page.

Close
