# Supplementary material for: Chromosome-level genome assembly and annotation of the loquat (Eriobotrya japonica) genome
Source: Gigascience. 2020 Mar 6;9(3):giaa015. doi: 10.1093/gigascience/giaa015 (PMC7059265; doi:10.1093/gigascience/giaa015)
Supplement: giaa015_Reviewer_1_Report_Original_Submission — Robert van Buren -- 11/9/2019 Reviewed [file giaa015_reviewer_1_report_original_submission.pdf]

Reviewer Report

Title: Chromosome-level genome assembly and annotation of the loquat (Eriobotrya japonica) genome

Version: Original Submission **Date:** 11/9/2019

Reviewer name: Robert van Buren

Reviewer Comments to Author:

Jiang et. al report a chromosome scale genome assembly of the important horticultural crop loquat. They utilized a single-molecule, Nanopore sequencing based approach coupled with Hi-C to generate a high-quality assembly. Loquat and apple have a clear2:2 synteny with a high degree of collinearity, suggesting they have a shared whole genome duplication event and that the loquat genome is high-quality. The resources presented here will be useful for the loquat and Rosaceae research communities as well as the comparative genomics communities. I have a few comments/suggestions that I feel with strengthen the manuscript.

Major:

1. Significantly more details are needed for the genome assembly section. Based on the methods, it seems like Canu was used to error correct the reads and smartdenovo assembly was used to assemble them into contigs. Then, Racon and Pilon were used to polish the assembly. This is an unusual pipeline to use and it is unclear which data was used in each step. Was the full Canu pipeline used to assemble a draft genome prior to smartdenovo assembly? Or, were error corrected or corrected and trimmed reads used as input for smartdenovo? Pilon requires Illumina short reads for polishing, was the HiSeq4000 data used for this? How many rounds of Racon and Pilon were run on the data? Statistics for each step of the assembly would also be helpful (e.g. how many errors were corrected, the input metrics for smartdenovo, etc.).
2. The manuscript contains would benefit from heavy editing for clarity.

Minor:

1. Line 65: "The reads were searched by the NT database, which confirmed that the sample is free from contamination."
It is unclear what this means. My interpretation is that a subset of reads were queried against a database using BLAST or another alignment program to identify contaminant sequences. More details should be provided here.
2. Line 82. A heterozygosity rate of 0.48 may be low relative compared to other highly heterozygotic species, but it would likely still present a challenge for genome assembly. Smartdenovo assembly will smash haplotypes together but programs like Canu should keep them separate during assembly. Was the full Canu pipeline run on the assembly? If so, how does this compare to the Smartdenovo assembly?
3. Line 87 Interrupted to sheared
4. Line 92. A protocol should be referenced for the HiC library construction
5. Line 106. Parameters should be reported for aligning the HiC reads to the genome using BWA.
6. Line 111. Interrupted to split

7. Line 116. It is unclear how 800 contigs were mapped to 17 chromosomes, but only 305 were oriented into the 17 pseudomolecules.
8. Line 148. More details should be provided on how transcripts were assembled and what cutoffs were used. Hisat and Stringtie are listed, but no details are provided.
9. Line 196. Loquat and apple have clear 2:2 synteny and shared Ks peaks, but it is not explicitly mentioned that they share a common whole genome duplication event.

Level of Interest

Please indicate how interesting you found the manuscript: Choose an item.

Quality of Written English

Please indicate the quality of language in the manuscript: Choose an item.

Declaration of Competing Interests

Please complete a declaration of competing interests, considering the following questions:

- Have you in the past five years received reimbursements, fees, funding, or salary from an organisation that may in any way gain or lose financially from the publication of this manuscript, either now or in the future?
- Do you hold any stocks or shares in an organisation that may in any way gain or lose financially from the publication of this manuscript, either now or in the future?
- Do you hold or are you currently applying for any patents relating to the content of the manuscript?
- Have you received reimbursements, fees, funding, or salary from an organization that holds or has applied for patents relating to the content of the manuscript?
- Do you have any other financial competing interests?
- Do you have any non-financial competing interests in relation to this paper?

If you can answer no to all of the above, write 'I declare that I have no competing interests' below. If your reply is yes to any, please give details below.

I declare that I have no competing interests

I agree to the open peer review policy of the journal. I understand that my name will be included on my report to the authors and, if the manuscript is accepted for publication, my named report including any attachments I upload will be posted on the website along with the authors' responses. I agree for my report to be made available under an Open Access Creative Commons CC-BY license (http://creativecommons.org/licenses/by/4.0/). I understand that any comments which I do not wish to

be included in my named report can be included as confidential comments to the editors, which will not be published.

Choose an item.

To further support our reviewers, we have joined with Publons, where you can gain additional credit to further highlight your hard work (see: https://publons.com/journal/530/gigascience). On publication of this paper, your review will be automatically added to Publons, you can then choose whether or not to claim your Publons credit. I understand this statement.

Yes Choose an item.
