# Supplementary material for: Chromosome-level genome assembly and annotation of the loquat (Eriobotrya japonica) genome
Source: Gigascience. 2020 Mar 6;9(3):giaa015. doi: 10.1093/gigascience/giaa015 (PMC7059265; doi:10.1093/gigascience/giaa015)
Supplement: giaa015_Reviewer_2_Report_Original_Submission — Jian-Feng Mao, Ph.D. -- 11/24/2019 Reviewed [file giaa015_reviewer_2_report_original_submission.pdf]

Reviewer Report

Title: Chromosome-level genome assembly and annotation of the loquat (Eriobotrya japonica) genome

Version: Original Submission **Date:** 11/24/2019

Reviewer name: Jian-Feng Mao, Ph.D.

Reviewer Comments to Author:

Jiang et al. reported the high quaility genome assembly and annotation for an important fruit tree, Eriobotrya japonica. In my opinion, this study is original, and data analysis have been well planned and conducted. The genomic resources and analysis are valuable for the loquat community and more broader regime of genomics and plant biology. However, there are large spaces for improvement in the English expression. I think editing by a native speaker is necessary.
It could be accepted after minor revision.
Major concern:
Please provide parameters and settings for specific analysis you conducted, especially for the genome assembly part.
Minor comments:
(I am not a native speaker. Here, I pick up specific comments related to generally the language expression)
1. line 11, "It flowered", is it a good expression?
2. line 19-21, please check this sentence, "The Hi-C ,,,, 39.7 Mb". Do you think Hi-C technology could really do assembly?
3. line 22, "analyzed" -> "investigated"
4. line 23, "the other six Rosaceae" -> "six other Rosaceae"
5. line 33, "are classified by" -> "were identified by"
6. line 34, "were" -> "are"
7. line 40, "The top buds become flowers", why do you want to say this?
8. line 41, why do you want to use "flowered"?
9. line 66-67, "Evaluation of the chloroplast of the species ,,, content", why do you want to do that? How did you do that? Is it relevant?
10. line 82, hard to imagine a simple genome, please define it if you want to describe your assembly as a simple one.
11. lines 91-92, "A Hi-C ,,, of the loquat", problematic expression. Do you really think the fresh leaf can do Hi-C? Please carefully check the full paper, for this similar problem.
12. line 94, "by" -> "with".
13. any reference for "smartdenovo"?
14. line 116, "assembled" -> "assembly"
15. no need to use "software" so often.
16. line 173, please define "unifamiles". and any typo there.

17. line 176, if you reported results of gene expansion. Please describe how you did do that?
18. line 209-210, "One-to-one corresponding chromosome", hard to explain this. Please define it or make it clear.
19. line 216, "Discussion" -> "Conclusion"
20. line 258, please carefully check your reference list. A lot bugs.
21. line 345, a new title would be "Picture of a loquat variety, seventh star"
22. Table 1 and 2, could be moved to the supplementary.
23. Replace Table 3 with Table S3.
24. line 384, please define "Uni family"
25. Figure 4, make it clear, by define the items you used or any other means.

Level of Interest

Please indicate how interesting you found the manuscript: Choose an item.

Quality of Written English

Please indicate the quality of language in the manuscript: Choose an item.

Declaration of Competing Interests

Please complete a declaration of competing interests, considering the following questions:

- Have you in the past five years received reimbursements, fees, funding, or salary from an organisation that may in any way gain or lose financially from the publication of this manuscript, either now or in the future?
- Do you hold any stocks or shares in an organisation that may in any way gain or lose financially from the publication of this manuscript, either now or in the future?
- Do you hold or are you currently applying for any patents relating to the content of the manuscript?
- Have you received reimbursements, fees, funding, or salary from an organization that holds or has applied for patents relating to the content of the manuscript?
- Do you have any other financial competing interests?
- Do you have any non-financial competing interests in relation to this paper?

If you can answer no to all of the above, write 'I declare that I have no competing interests' below. If your reply is yes to any, please give details below.

no

I agree to the open peer review policy of the journal. I understand that my name will be included on my report to the authors and, if the manuscript is accepted for publication, my named report including any

attachments I upload will be posted on the website along with the authors' responses. I agree for my report to be made available under an Open Access Creative Commons CC-BY license (http://creativecommons.org/licenses/by/4.0/). I understand that any comments which I do not wish to be included in my named report can be included as confidential comments to the editors, which will not be published.

Choose an item.

To further support our reviewers, we have joined with Publons, where you can gain additional credit to further highlight your hard work (see: https://publons.com/journal/530/gigascience). On publication of this paper, your review will be automatically added to Publons, you can then choose whether or not to claim your Publons credit. I understand this statement.

Yes Choose an item.
