# Supplementary material for: Chromosome-level genome assembly and annotation of the loquat (Eriobotrya japonica) genome
Source: Gigascience. 2020 Mar 6;9(3):giaa015. doi: 10.1093/gigascience/giaa015 (PMC7059265; doi:10.1093/gigascience/giaa015)
Supplement: giaa015_Reviewer_3_Report_Original_Submission — Soichiro Nagano -- 11/28/2019 Reviewed [file giaa015_reviewer_3_report_original_submission.pdf]

**Reviewer Report**

**Title: Chromosome-level genome assembly and annotation of the loquat (Eriobotrya japonica) genome**

**Version: Original Submission** **Date:** 11/28/2019

**Reviewer name: Soichiro Nagano**

**Reviewer Comments to Author:**

This paper is worth publishing for the Data Note for GigaScience because the authors have constructed a highly accurate genome and gene sequence of loquat. The method is reasonable and the presentation is pretty good. Speaking of greed, since it is clear that the relationship with Malus is relatively close among the fruit trees of the Rosaceae family, so there should be presented some discussion about the traces on the genome that triggered the differentiation of morphological features between both species, I recommend that as it would be done for the future work. The following minor concerns should be corrected before acceptance.

Minor concerns

L58 How to extract the RNA from the collected samples should be described.

L63 double-end would be pair-end.

L71 the appearance of the equation is not clear. Please correct the format of the equation.

L86 "quality" have to be "quantity", because the Qubit 2.0 Fluorometer is the device to evaluate the quantity of the DNA/RNA with fluorescence.

L89 How many flow cells have been used with the PromethION platform to acquire about the 106.23 Gb.

L205 "Sac1, 4, and 8" has been suddenly appeared at the text. Please describe about relationships between the former and later text to be clearly understood, although chromosome scale duplication is very interesting.

L349 "de novo" should be written in italic.

L364 Add period.

L366 Add period.

L367 E. japonica should be written in italic.

L375 "Hic" should be "Hi-C"

Figures Provide higher resolutional figures than current version. Because those are not clear.

Figure 4A Correct the overlap of the legends on a bar.

Figure 5A It is difficult to see the scale of the figure. Please provide higher resolutional figures.

**Level of Interest**

Please indicate how interesting you found the manuscript: Choose an item.

**Quality of Written English**

Please indicate the quality of language in the manuscript: Choose an item.

**Declaration of Competing Interests**

Please complete a declaration of competing interests, considering the following questions:

- Have you in the past five years received reimbursements, fees, funding, or salary from an organisation that may in any way gain or lose financially from the publication of this manuscript, either now or in the future?
- Do you hold any stocks or shares in an organisation that may in any way gain or lose financially from the publication of this manuscript, either now or in the future?
- Do you hold or are you currently applying for any patents relating to the content of the manuscript?
- Have you received reimbursements, fees, funding, or salary from an organization that holds or has applied for patents relating to the content of the manuscript?
- Do you have any other financial competing interests?
- Do you have any non-financial competing interests in relation to this paper?

If you can answer no to all of the above, write 'I declare that I have no competing interests' below. If your reply is yes to any, please give details below.

I declare that I have no competing interests.

I agree to the open peer review policy of the journal. I understand that my name will be included on my report to the authors and, if the manuscript is accepted for publication, my named report including any attachments I upload will be posted on the website along with the authors' responses. I agree for my report to be made available under an Open Access Creative Commons CC-BY license (http://creativecommons.org/licenses/by/4.0/). I understand that any comments which I do not wish to be included in my named report can be included as confidential comments to the editors, which will not be published.

Choose an item.

To further support our reviewers, we have joined with Publons, where you can gain additional credit to further highlight your hard work (see: https://publons.com/journal/530/gigascience). On publication of this paper, your review will be automatically added to Publons, you can then choose whether or not to claim your Publons credit. I understand this statement.

Yes Choose an item.
